# Association between oxygen saturation ranges and mortality among mechanically ventilated sepsis-associated ARDS patients: a multicenter retrospective cohort analysis

**DOI:** 10.3389/fmed.2026.1862391

**Published:** 2026-08-21

**Authors:** Bojun Li, Yu Meng, Xiang Pei, Bo Li, Xiaoyun Zhao

**Affiliations:** 1 Department of Pulmonary and Critical Care Medicine, Tianjin Chest Hospital, Tianjin, China; 2 Department of Critical Care Medicine, Tianjin Medical University General Hospital, Tianjin, China

**Keywords:** ARDS, critical care, mechanical ventilation, oxygen saturation, sepsis

## Abstract

**Background:**

The SpO_2_ range associated with the best survival outcomes for acute respiratory distress syndrome (ARDS), particularly in sepsis-related cases, remains unclear. This study aimed to explore oxygenation range for patients with sepsis and ARDS during mechanical ventilation.

**Methods:**

We analyzed 2951 ICU patients with sepsis and ARDS requiring mechanical ventilation. Using restricted cubic splines (RCS) and generalized additive models (GAM), we identified optimal oxygenation ranges, which were further cross-replicated across independent ICU datasets via logistic regression, propensity score matching, IPW, and double robustness analysis. The primary outcome was 28-day all-cause mortality.

**Results:**

In this cohort, the range of oxygen saturation (SpO_2_) with 93%–99% was associated with the lowest 28-day mortality in patients with sepsis and ARDS, as determined by RCS and GAMs. These findings were verified via multivariate logistic regression, propensity score matching, IPW, and double-robust adjustment incorporating all available covariates. This range SpO_2_ window remained consistent across mild, moderate and severe ARDS subgroups. Patients with SpO_2_ < 93% had higher SOFA, APACHE II and SAPS II scores. Infection source did not significantly modify the SpO_2_-mortality association.

**Conclusion:**

SpO_2_ ranging from 93% to 99% was independently associated with reduced 28-day all-cause mortality in mechanically ventilated patients with sepsis-related ARDS in this retrospective cohort.

## Background

Patients with sepsis account for 20%–30% of ICU patients.(1) The mortality rate for sepsis combined with organ dysfunction is as high as 52.3%.(2) Acute respiratory distress syndrome (ARDS) is a common sepsis-associated organ injury,(3) with mild, moderate, and severe cases of ARDS having mortality rates of 34.9%, 40.3%, and 46.1%, respectively (4). This syndrome appears to be under-recognized and untreated, contributing to its high mortality rate and indicating a potential for improving the management of patients with ARDS.

Patients with sepsis and ARDS are typically treated using mechanical ventilation.(5) Low tidal volume and high positive end-expiratory pressure (PEEP) ventilation can improve the prognosis of patients with ARDS.(6) However, the range of oxygenation value for patients with ARDS remains unclear. Low blood oxygenation predisposes patients to tissue hypoxia and, eventually, cell death. Conversely, high blood oxygenation may induce vasoconstriction, and high inspired oxygen concentrations can cause direct pulmonary toxicity and atelectasis (7). Therefore, the relationship between oxygenation and pulmonary disease outcomes may be non-linear. However, randomized trials have not identified the range of oxygenation that affect overall outcomes in patients with ARDS (8). A recent study found that individualized oxygenation range can reduce mortality in critically ill adults, with higher oxygen saturation (SpO_2_) in patients with sepsis associated with more severe conditions.(9) The range blood oxygen value for patients with sepsis appears to have a non-linear relationship. Previous randomized controlled trials (RCTs) on the optimal SpO_2_ for ARDS have addressed various factors contributing to ARDS.(8) Based on recent research findings, individualized oxygenation range for critically ill adults are beneficial; however, the clinically appropriate oxygen saturation range and its causal impact on ARDS prognosis remain incompletely defined.

This study explored the relationship between oxygenation indicators and the prognosis of patients with sepsis and ARDS in the ICU of Tianjin Chest Hospital. We further cross-replicated our optimal oxygenation findings using two independent U.S. ICU datasets: the MIMIC-IV and eICU-CRD databases, to strengthen the generalizability of our single-center observations.

## Materials and methods

### Data description

The study cohort was obtained from the Department of Pulmonary and Critical Care Medicine of Tianjin Chest Hospital between January 2023 and January 2026. The external validation cohort was sourced from the MIMIC IV database and eICU-CRD. The study protocol for patients with sepsis and ARDS at Tianjin Chest Hospital was approved by the hospital’s Ethics Committee (IRB: 2026LW-023). The MIMIC IV v2.2 and eICU-CRD v2.0 were approved by the Institutional Review Board of the Beth Israel Deaconess Medical Center (2001-P001699/14) and the Massachusetts Institute of Technology (No. 0403000206). The eICU-CRD includes data from 200,859 ICU admissions of 139,367 patients across 208 U.S. hospitals from 2014 to 2015 (10). The MIMIC IV database contains clinical data for over 190,000 patients admitted to Beth Israel Deaconess Medical Center in Boston between 2008 and 2019 (11).

### Patients

The patients in this study met the following criteria: they had a diagnosis of sepsis 3.0, (12) were diagnosed with ARDS according to the Berlin criteria, (13) were 18 years old or older, and had an ICU stay of more than 24 h. The exclusion criteria included having congestive heart failure, cardiogenic pulmonary edema, extensive atelectasis, alveolar hemorrhage, extensive pleural effusion, acute onset of idiopathic interstitial lung disease, and extensive pulmonary embolism. Patients with SpO_2_ measurements taken within less than 24 h; those who had not been placed on a ventilator to assist breathing, and those with missing values for important indicators such as PEEP, fraction of inspiration O_2_ (FiO_2_), SpO_2_, and respiratory rate (RR) were also excluded.

### Definition of key clinical time points

We standardized operational definitions for sepsis onset, ARDS onset, mechanical ventilation initiation, and oxygenation measurement timing across the local single-center cohort, MIMIC IV, and eICU-CRD databases:

#### Sepsis onset

Tianjin Chest Hospital cohort, MIMIC IV and eICU-CRD: Sepsis was defined as meeting Sepsis-3 criteria (suspected infection + SOFA ≥ 2) at ICU admission, with diagnostic timestamps extracted from EMR and database records.

#### ARDS onset

Consistent with the Berlin definition, ARDS onset was restricted to cases with respiratory failure developing within 7 days after the sepsis. Tianjin Chest Hospital cohort: ARDS onset was recorded as the first time point meeting Berlin oxygenation and radiographic criteria on ventilator support. MIMIC IV and eICU-CRD: ARDS onset was defined as the earliest timestamp satisfying all Berlin ARDS criteria recorded in structured ICU tables, including hypoxemia categorized by PaO_2_/FiO_2_ ratio, bilateral pulmonary infiltrates on chest imaging, and respiratory failure not fully explained by cardiac failure and others. A 7-day onset window relative to ICU admission sepsis diagnosis was strictly implemented during data filtering.

#### Initiation of mechanical ventilation

Tianjin Chest Hospital cohort: The exact time of invasive mechanical ventilation setup recorded in ICU nursing records. MIMIC IV and eICU-CRD: Extracted from ventilation device chart tables, defined as the start time of continuous invasive ventilator support.

#### Oxygenation measurement window

All SpO_2_, PaO_2_, FiO_2_ and respiratory rate measurements were captured every 8 h after mechanical ventilation initiation, up to a maximum of 7 days of ventilation. Only patients with valid oxygenation data recorded ≥24 h after ventilation start were retained for analysis, consistent with our exclusion criteria. Oxygenation indices were aggregated as median values across the full observation window for primary analyses.

We unified time zero (ICU Sepsis-3 admission) and oxygen exposure windows across cohorts. Oxygen sampling began at ventilation initiation with fixed 8-h intervals up to day 7; only patients with ≥24 h valid readings were included to avoid immortal time bias. Serial oxygen metrics were summarized as patient median SpO_2_ for static exposure classification, consistently applied to all subgroups.

### Data extraction

The prespecified primary outcome was 28-day all-cause mortality in mechanically ventilated patients with sepsis-associated ARDS following ICU admission. 90-day all-cause mortality was predefined as a secondary exploratory endpoint for long-term sensitivity analysis, used to confirm whether the association between oxygen saturation ranges and survival persisted beyond day 28. All 90-day mortality analyses were secondary sensitivity analyses supporting the primary 28-day mortality findings. Oxygen exposure parameters including SpO_2_, PaO_2_, FiO_2_ and respiratory rate (RR) were documented every 8 h after mechanical ventilation initiation, with data collection capped at day 7 of ventilation for patients receiving prolonged ventilatory support, and terminated upon extubation for patients with shorter ventilation duration. For all primary spline, regression, propensity score-based and survival analyses, oxygenation indices were aggregated as the median value of all valid serial measurements recorded throughout the entire mechanical ventilation observation window to generate a single summary oxygenation metric per patient. All SpO_2_ measurements were captured every 8 h after mechanical initiation, capped at day 7 (maximum 21 serial measurements per patient). For all primary analyses, a single patient-level representative SpO_2_ value was computed as the median of all valid serial SpO_2_ readings recorded throughout the entire ventilation period. Patients were dichotomized into the 93%–99% SpO_2_ group if their patient-level median SpO_2_ fell within this range; sustained SpO_2_ within the range window at every time point was not required, and isolated transient out-of-range readings did not alter patient grouping. Baseline data included age, sex, coexisting illnesses, site of infection, pathogenic organism, length of ICU stay, renal replacement therapy, duration of mechanical ventilation, use of vasoactive drugs or inotropic support, and 28-day and 90-day mortality. Additionally, we collected Sequential Organ Failure Assessment (SOFA), Acute Physiology and Chronic Health Evaluation (APACHE II), and Simplified Acute Physiology Score II (SAPS II) scores within 24 h of ICU admission. The median vital signs and laboratory test results of the patients were also collected during mechanical ventilation.

### Statistical analysis

Shapiro–Wilk tests confirmed all continuous variables followed skewed distributions. Data are presented as the interquartile range (IQR). Differences between survivors and non-survivors were analyzed using Wilcoxon rank-sum and Fisher’s exact tests. Generalized additive models (GAM) (14) and restricted cubic spline (RCS) (15) were used to explore the relationships between SpO_2_, PaO_2_, PaO_2_/FiO_2_, SpO_2_/FiO_2_, and 28-day and 90-day mortality. For the eICU-CRD data, we included the hospital as a random intercept to capture the correlation between cases from the same hospital while mitigating biases due to differences between hospitals. The results from the GAM and RCS, adjusted for other influencing factors, showed a non-linear relationship between SpO_2_ and PaO_2_ in patients with sepsis and ARDS. Beyond patient-level median SpO_2_ as static oxygen exposure summary, we further calculated two time-integrated oxygen metrics to quantify cumulative oxygen burden per mechanical ventilation episode, time-weighted average SpO_2_ (TWA-SpO_2_) and area under the SpO_2_-time curve (SpO_2_-AUC). Serial SpO_2_ measurements were recorded every 8 h from ventilation initiation up to day 7; linear trapezoidal integration was applied to compute SpO_2_-AUC across all valid 8-h intervals within the observation window. TWA-SpO_2_ was defined as the time-weighted mean saturation weighted by the duration between consecutive SpO_2_ recordings. RCS models adjusted for identical baseline confounders (SOFA, APACHE II, cancer history, mechanical ventilation duration, vasoactive/inotropic support) were constructed to visualize the continuous non-linear association between TWA-SpO_2_/SpO_2_-AUC and 28-day all-cause mortality in the Tianjin Chest Hospital cohort. Within the single-center Tianjin Chest Hospital cohort, we conducted multiple internal sensitivity analyses including multivariate logistic analysis, Kaplan–Meier curves, propensity score matching, inverse probability weighting (IPW), and double-robust statistical methods (16, 17) to test the stability of associations. We replicated identical analytical frameworks in the MIMIC-IV and eICU-CRD cohorts to evaluate consistent associations across distinct ICU datasets. The IPW was calculated based on the propensity score-matching weight to reduce the imbalance in the distribution of covariates. The doubly robust estimation model combines the multivariate logistic and propensity score matching models to obtain an effect estimator with double robustness. Our principled adjustment set only incorporated baseline covariates obtained upon ICU admission. The standardized mean difference (SMD) was used to assess the quality of the propensity score matching. If the SMD does not exceed 0.1, the matching quality for this variable is generally considered acceptable. In this study, the SMDs for Tianjin Chest Hospital, the MIMIC IV database, and the eICU database were all less than 0.1 (Supplementary eFigures 1–3). Core respiratory metrics including SpO_2_, FiO_2_, PEEP and respiratory rate were extracted for all patients. Nevertheless, tidal volume, plateau pressure, driving pressure and prone positioning were excluded from multivariable regression, propensity score matching, IPW and double robustness analyses due to substantial missing data and heterogeneous documentation across the three cohorts. Notably, PEEP was routinely recorded but not incorporated into the primary covariate adjustment set.

Stratified analyses by ARDS severity (mild, moderate, severe, defined by Berlin PaO_2_/FiO_2_ thresholds) were performed to assess heterogeneity in the SpO_2_–28-day mortality association. Separate restricted cubic spline models and fully adjusted logistic regression models were fitted within each severity subgroup, with effect estimates and 95% CIs reported. To test whether infection source (pulmonary vs. non-pulmonary infection) modified the association between SpO_2_ category and 28-day mortality, an interaction term of SpO_2_ group × infection source was incorporated into the multivariate logistic regression model; interaction *P*-values were calculated to judge statistical effect modification.

For missing data handling: Patients with missing core respiratory indicators (SpO_2_, FiO_2_, PEEP, RR) over the 24 h mechanical ventilation monitoring window were directly excluded per predefined eligibility criteria. We further quantified missingness rates of all key covariates, baseline laboratory markers, and secondary endpoints across three cohorts (Tianjin Chest Hospital, MIMIC IV, eICU-CRD). A total of 1210 patients were excluded due to missing SpO_2_, FiO_2_, PEEP, RR core respiratory indicators, accounting for 10.38% of all initially screened sepsis-ARDS candidates. All non-outcome variables with incomplete records were processed via multiple imputation (MI) with 20 imputed datasets, while primary (28-day all-cause mortality) and secondary (90-day mortality) outcome variables contained zero missing values and thus required no imputation. Missing proportions of critical variables were summarized in Supplementary eTable 1. The above statistical analyses were performed by R Studio software. *P* < 0.05 was considered statistically significant.

## Results

### Baseline characteristics of the patients

A total of 2951 patients with sepsis met the ARDS Berlin diagnostic criteria. According to the inclusion and exclusion criteria, there were 1022 patients from the Tianjin Chest Hospital and MIMIC IV databases, and 907 patients from the eICU database (Supplementary eFigure 4).

Table 1 and Supplementary eTables 2, 3 summarize the demographic and clinical characteristics of the Tianjin Chest Hospital cohort. Among patients with sepsis-associated ARDS, those complicated with metastatic cancer, *Acinetobacter baumannii*, or *Klebsiella pneumoniae* infection exhibited higher 28-day and 90-day all-cause mortality. Among patients with sepsis-associated ARDS, lower SpO_2_, PaO_2_, PaO_2_/FiO_2_ and SpO_2_/FiO_2_ levels were associated with higher 28-day and 90-day all-cause mortality, whereas higher FiO_2_ levels also predicted increased mortality. Supplementary eTables 2, 3 shows that non-surviving patients with sepsis and ARDS had worse hemodynamic parameters and tissue perfusion, coagulation function, liver and kidney function, and more severe infections.

**TABLE 1:** Baseline and outcome of sepsis patients and ARDS.

Characteristic	Survivor (*n* = 587)	Non-survivor (*n* = 435)	*P*
Age, years	68.00 [55.00, 75.00]	67.00 [55.00, 75.00]	0.429
Male sex, *n* (%)	240 (40.9)	153 (35.2)	0.073
Site of infection, n (%)			
Pulmonary	512 (87.2)	377 (86.7)	0.867
Abdominal	132 (22.5)	83 (19.1)	0.214
Urinary	54 (9.2)	23 (5.3)	0.026
Skin soft tissue	24 (4.1)	15 (3.4)	0.716
Catheter	29 (4.9)	26 (6.0)	0.558
Pathogenic microorganisms, n (%)			
*Acinetobacter baumannii*	89 (15.2)	112 (25.7)	<0.001
*Klebsiella pneumoniae*	112 (19.1)	134 (30.8)	<0.001
*Pseudomonas aeruginosa*	80 (13.6)	40 (9.2)	0.038
*Escherichia coli*	55 (9.4)	30 (6.9)	0.193
*Staphylococcus aureus*	61 (10.4)	51 (11.7)	0.567
Respiration			
Respiratory rate, bpm	23.0 [18.0, 29.0]	22.0 [17.0, 29.00]	0.123
SpO_2_, %	97.00 [94.00, 98.00]	93.00 [92.00, 94.00]	<0.001
PaO_2_, mmHg	94.20 [88.35, 107.10]	73.30 [65.90, 80.45]	<0.001
PaCO_2_, mmHg	38.00 [31.80, 42.80]	38.00 [32.00, 44.10]	0.683
FiO_2_	0.60 [0.50, 0.60]	0.70 [0.50, 0.80]	<0.001
PaO_2_/FiO_2_ ratio	169.90 [142.40, 197.60]	111.60 [97.20, 154.35]	<0.001
SpO_2_/FiO_2_ ratio	171.00 [154.20, 188.25]	127.80 [112.30, 172.20]	<0.001
PEEP, cmH_2_O	7.20 [6.60, 7.60]	8.00 [7.70, 8.30]	<0.001
ARDS-severity			<0.001
Mild	130 (22.15)	80 (18.4)	
Moderate	450 (76.6)	222 (51.0)	
Severe	7 (1.3)	133 (30.6)	
Therapy, n (%)			
Prone ventilation therapy	20 (3.4)	19 (4.4)	0.53
Use of glucocorticoids	122 (20.8)	86 (19.8)	0.749
Use of muscle relaxant	17 (2.9)	14 (3.2)	0.91
Outcome			
ICU length of stay, (days)	15.00 [8.63, 28.73]	9.91 [4.26, 18.00]	<0.001
Inotropic/vasopressor support, *n* (%)	346 (58.9)	295 (67.8)	0.005
Duration of mechanical ventilation, (days)	8.00 [3.77, 18.00]	8.00 [3.00, 13.13]	0.004
Renal replacement therapy, *n* (%)	172 (29.3)	197 (45.3)	<0.001
APACHE II score	14.00 [13.00, 19.00]	24.00 [21.00, 27.00]	<0.001
SOFA score	8.00 [5.00, 8.00]	10.00 [8.00, 12.00]	<0.001

FiO_2_, fraction of inspiration O_2_; PaCO_2_, partial pressure of carbon dioxide; PaO_2_, partial pressure of oxygen; PEEP, positive end-expiratory pressure; APACHE II, Acute Physiology and Chronic Health Evaluation; SOFA, Sequential Organ Failure Assessment; SpO_2_, arterial oxygen saturation; the continuous variables were skewed and therefore described in the form of IQR.

### The outcome of the patients

Table 1 summarizes the outcomes of patients with sepsis and ARDS in the Tianjin Chest Hospital cohort. Patients in the non-survival group had higher SOFA and APACHE II scores, longer ICU stay, longer durations of mechanical ventilation, and more patients were treated with inotropic, vasopressor, or continuous renal replacement therapy (CRRT) support.

### The oxygen saturation range

**FIGURE 1:**
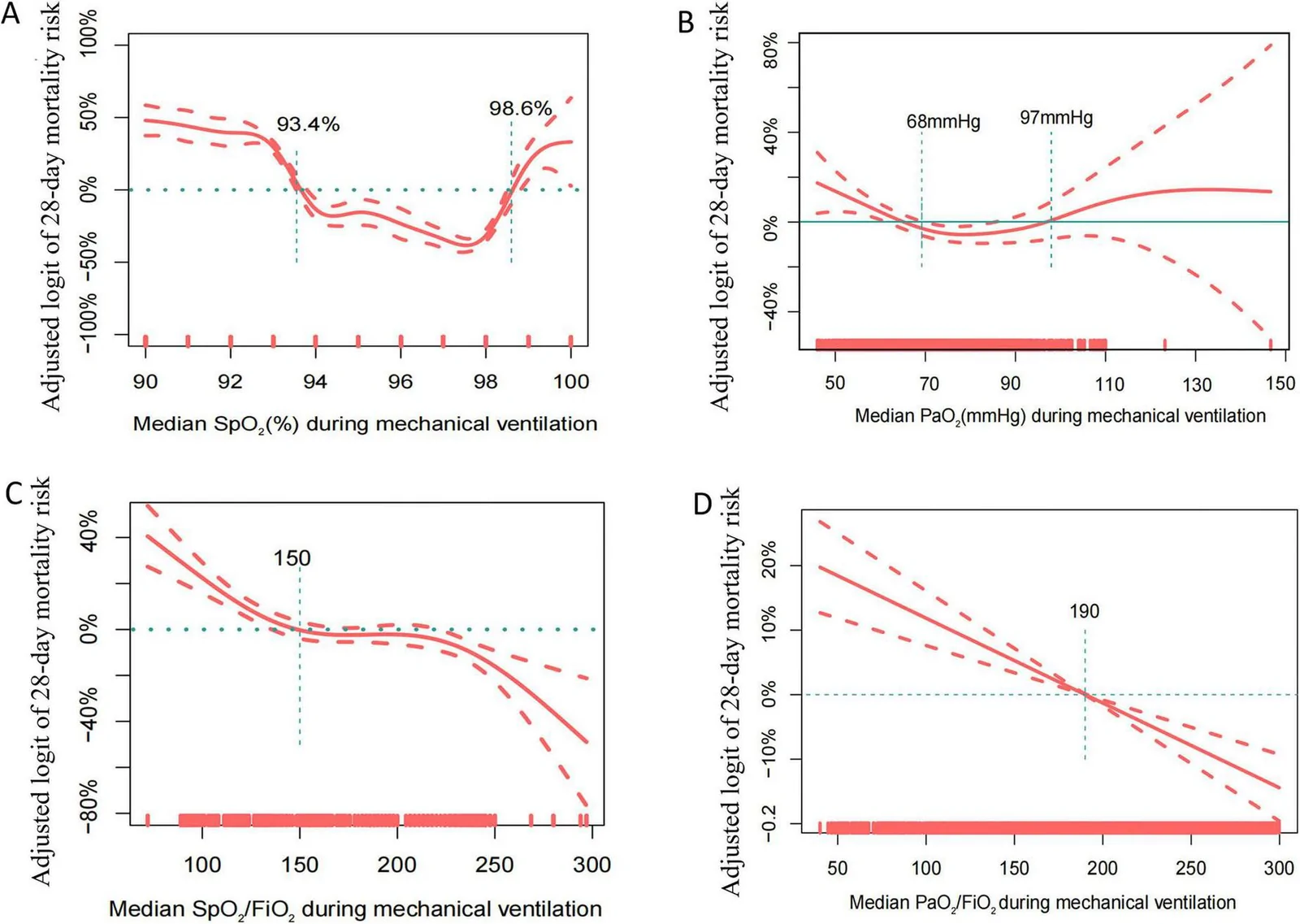
The relationship between the 28-day mortality of patients with sepsis and ARDS and median oxygenation indicators in the Tianjin Chest Hospital using a generalized additive model. **(A,B)** show that the SpO_2_ range was approximately 93–99% and the PaO_2_ range was approximately 68–97mmHg for patients with sepsis and ARDS. **(C,D)** show that SpO_2_/FiO_2_ and PaO_2_/FiO_2_ had a non-U-shaped relationship with the 28-day mortality of patients with sepsis and ARDS. Solid red lines = predicted mortality logit; dashed red lines = 95% confidence intervals; horizontal dotted line = baseline reference risk; vertical dashed lines = critical oxygen thresholds; *x*-axis rug ticks represent individual patient data distribution.

Figure 1 and Supplementary eFigure 3 show a U-shaped association between the median SpO_2_ (*P* < 0.001) and 28-day mortality and 90-day mortality in patients with sepsis and ARDS in the Tianjin Chest Hospital cohort using the GAM. Besides, Figure 1 and Supplementary eFigure 5 show a U-shaped association between the median PaO_2_ (*P* < 0.001) and prognosis in patients with sepsis and ARDS. The SpO_2_ range of 93%–99% was associated with the lowest 28-day mortality, and the PaO_2_ range was approximately 68–97 mmHg or 64–85 mmHg for these patients. Figure 1 and Supplementary eFigure 5 shows a non-U-shaped association between the median SpO_2_/FiO_2_, and PaO_2_/FiO_2_ 28-day and 90-day mortality in patients with sepsis and ARDS.

**FIGURE 2:**
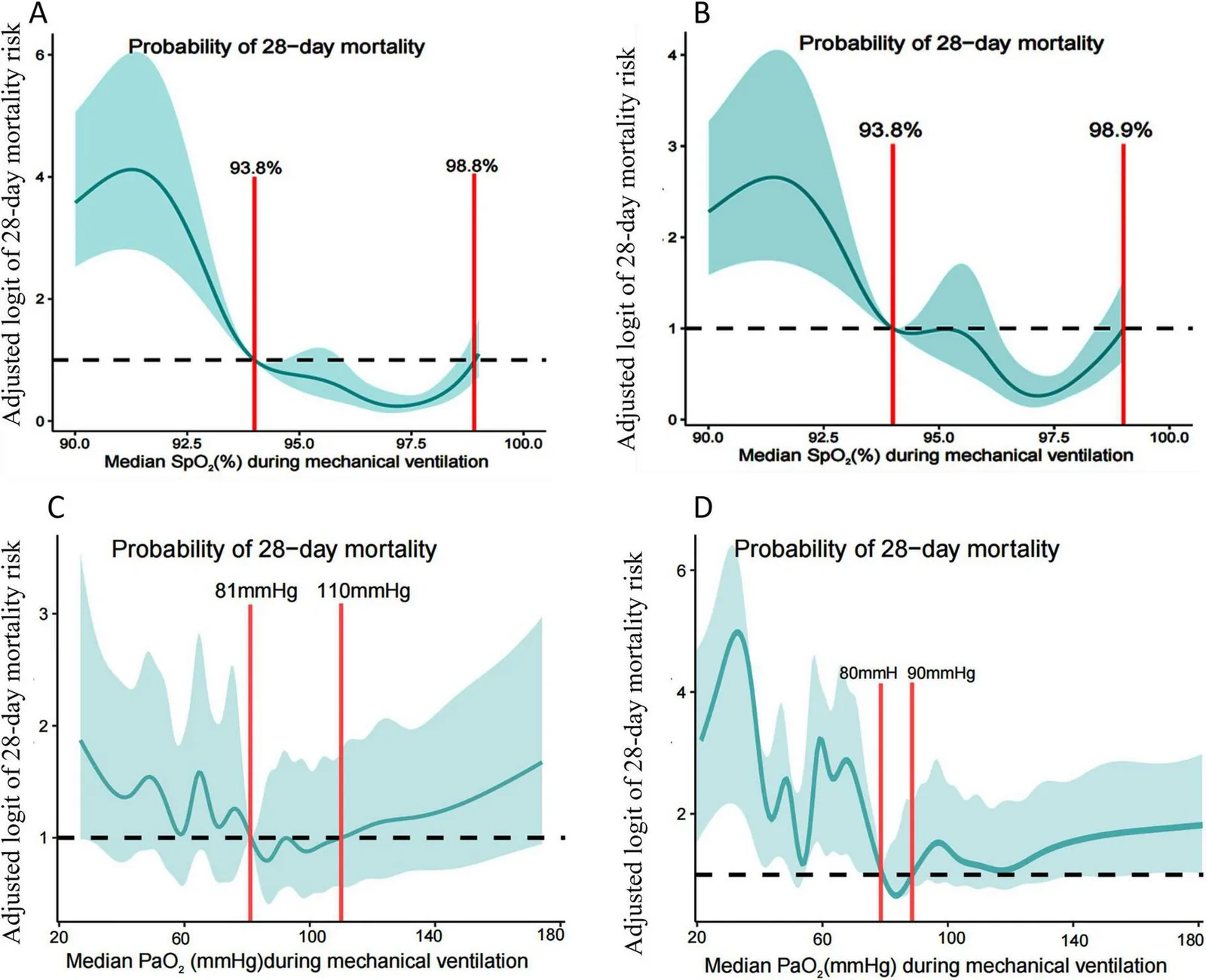
The relationship between the 28-day mortality of patients with sepsis and ARDS and median SpO_2_ and PaO_2_ in the Tianjin Chest Hospital using restricted cubic spline. **(A)** Shows that the SpO_2_ range was approximately 93%–99% for patients with sepsis and ARDS, without adjustment for other factors. **(B)** Adjusted for SOFA, APACHE II, history of cancer, and vasoactive or inotropic support, still supports the results shown in panel **(A)**. **(C)** Shows that the PaO_2_ range was approximately 81–110 mmHg for patients with sepsis and ARDS, without adjustment for other factors. **(D)** Adjusted for SOFA, APACHE II, history of cancer, and vasoactive or inotropic support, indicates that the PaO_2_ range was approximately 80–90 mmHg for patients with sepsis and ARDS. Solid lines = predicted mortality logit; shaded regions = 95% CIs; dashed horizontal line = baseline reference risk; vertical red lines = identified critical oxygen thresholds.

**FIGURE 3:**
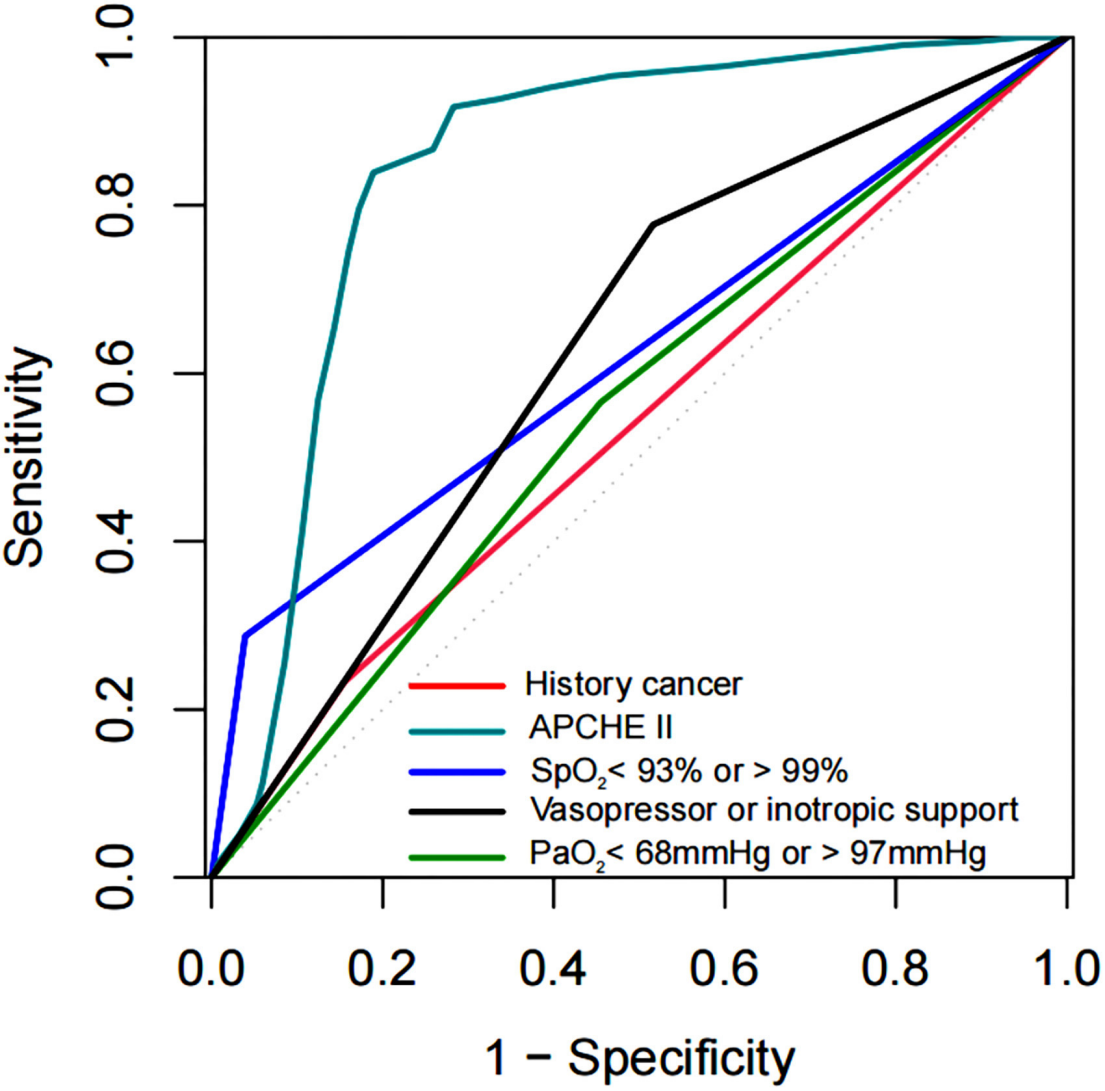
ROC curves for predicting 28-day all-cause mortality among mechanically ventilated patients with sepsis-associated ARDS from Tianjin Chest Hospital. *X*-axis = 1 - specificity; *Y*-axis = sensitivity. APACHE II, Acute Physiology and Chronic Health Evaluation II.

To verify the reliability of the results shown in Figure 1, an RCS was used. The results of Figure 2A, which were not adjusted for other factors, showed that the SpO_2_ range was approximately 93%–99% for patients with sepsis and ARDS. Figure 2B, adjusted for SOFA, APACHE II, history of cancer, duration of mechanical ventilation, and vasoactive or inotropic support, still supported the results shown in Figure 1. The results of Figure 2C, which were not adjusted for other factors, showed that the PaO_2_ range was approximately 81–110 mmHg for patients with sepsis and ARDS. Figure 2D, adjusted for SOFA, APACHE II, history of cancer, duration of mechanical ventilation, and vasoactive or inotropic support, indicated that the PaO_2_ range was approximately 80–90 mmHg for patients with sepsis and ARDS. The results of the study suggest that 68–97 mmHg PaO_2_ and 93%–99% SpO_2_ were independent correlates of favorable 28-day prognosis for the 28-day prognosis of patients with sepsis and ARDS, with good specificity and sensitivity (Figure 3, Table 2 and Supplementary eTables 4, 5).

**TABLE 2:** The multiple models analysis of SpO_2_ to 28 day mortality in sepsis patient and ARDS.

Models	HR	CI	*P*
		2.5% 97.5%	
Tianjin Chest Hospital			
Multivariate logistic analysis			
93% ≤ SpO_2_ ≤ 99%	0.56	0.44–0.71	<0.001
Propensity score matching			
93% ≤ SpO_2_ ≤ 99%	0.21	0.11- 0.37	<0.001
Propensity score IPW			
93% ≤ SpO_2_ ≤ 99%	0.11	0.06–0.21	<0.001
Doubly robust with all covariates			
93% ≤ SpO_2_ ≤ 99%	0.59	0.53–0.65	<0.001
MIMIC IV database			
Multivariate logistic analysis			
93% ≤ SpO_2_ ≤ 99%	0.65	0.44–0.95	0.043
Propensity score matching			
93% ≤ SpO_2_ ≤ 99%	0.88	0.64, 1.22	0.453
Propensity score IPW			
93% ≤ SpO_2_ ≤ 99%	0.67	0.46- 0.99	0.043
Doubly robust with all covariates			
93% ≤ SpO_2_ ≤ 99%	0.73	0.58–0.90	0.004
eICU database			
Multivariate logistic analysis			
93% ≤ SpO_2_ ≤ 99%	0.51	0.36–0.73	<0.001
Propensity score matching			
93% ≤ SpO_2_ ≤ 99%	0.68	0.52, 0.90	0.006
Propensity score IPW			
93% ≤ SpO_2_ ≤ 99%	0.55	0.39, 0.77	0.001
Doubly robust with all covariates			
93% ≤ SpO_2_ ≤ 99%	0.67	0.55, 0.82	<0.001

**FIGURE 4:**
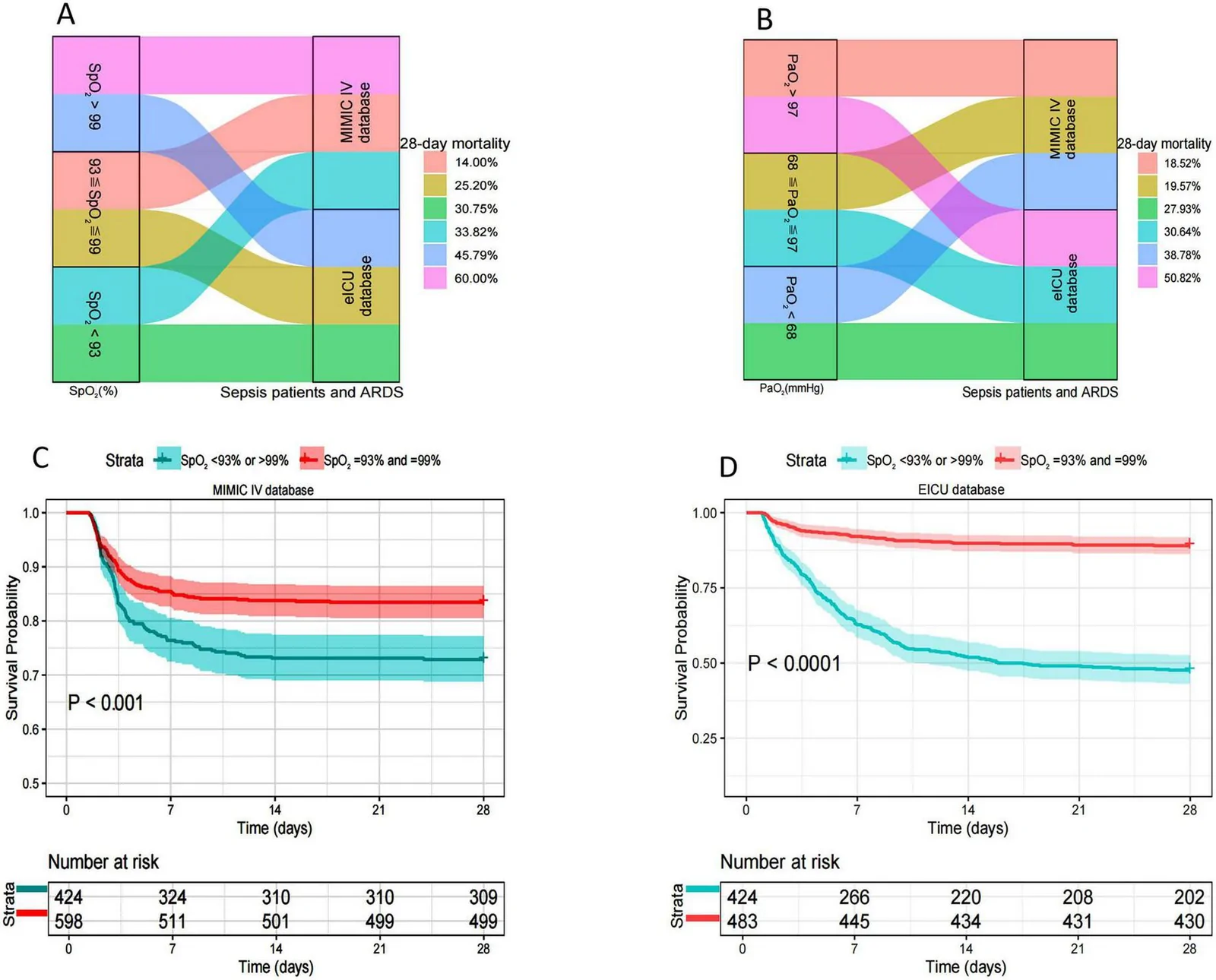
Cross-dataset replication of the associations between SpO_2_, PaO_2_ and 28-day all-cause mortality in mechanically ventilated patients with sepsis-associated ARDS. **(A)** Unadjusted association between median SpO_2_ and 28-day mortality in the Tianjin Chest Hospital single-center cohort; **(B)** unadjusted association between median PaO_2_ and 28-day mortality in the Tianjin Chest Hospital single-center cohort; **(C)** Kaplan–Meier survival curves stratified by SpO_2_ strata (SpO_2_ < 93%, 93%–99%, SpO_2_ > 99%) from the MIMIC IV database; **(D)** Kaplan–Meier survival curves stratified by SpO_2_ strata from the eICU-CRD database. For panels **(C,D)**, the *X*-axis denotes time days and the *Y*-axis denotes cumulative survival probability; *P* < 0.01 indicates significant inter-group survival differences between patients with SpO_2_ 93%–99% versus those outside this optimal range.

We replicated our analyses within the MIMIC-IV and eICU-CRD ICU datasets. Figure 4A showed that patients with sepsis and ARDS with SpO_2_ between 93% and 99% had a lower mortality rate compared to those with SpO_2_ < 93% or SpO_2_ > 99% in the Tianjin Chest Hospital cohort. Figure 4B showed that patients with sepsis and ARDS with PaO_2_ between 68 mmHg and 97 mmHg had a lower mortality rate compared to those with PaO_2_ < 68 mmHg or PaO_2_ > 97 mmHg in the Tianjin Chest Hospital cohort. Figures 4C,D demonstrate that patients with SpO_2_ between 93% and 99% had significantly better survival than that of SpO_2_ < 93% or SpO_2_ > 99% through the MIMIC IV database and the eICU database (*P* < 0.01). However, the survival rates for the range PaO_2_ levels for patients with sepsis and ARDS, whether 68–97 mmHg, 81–110 mmHg, or 80–90 mmHg, were not significantly different through the externally validated MIMIC IV database and the eICU database (*P* > 0.05) (Supplementary eFigures 6, 7).

**FIGURE 5:**
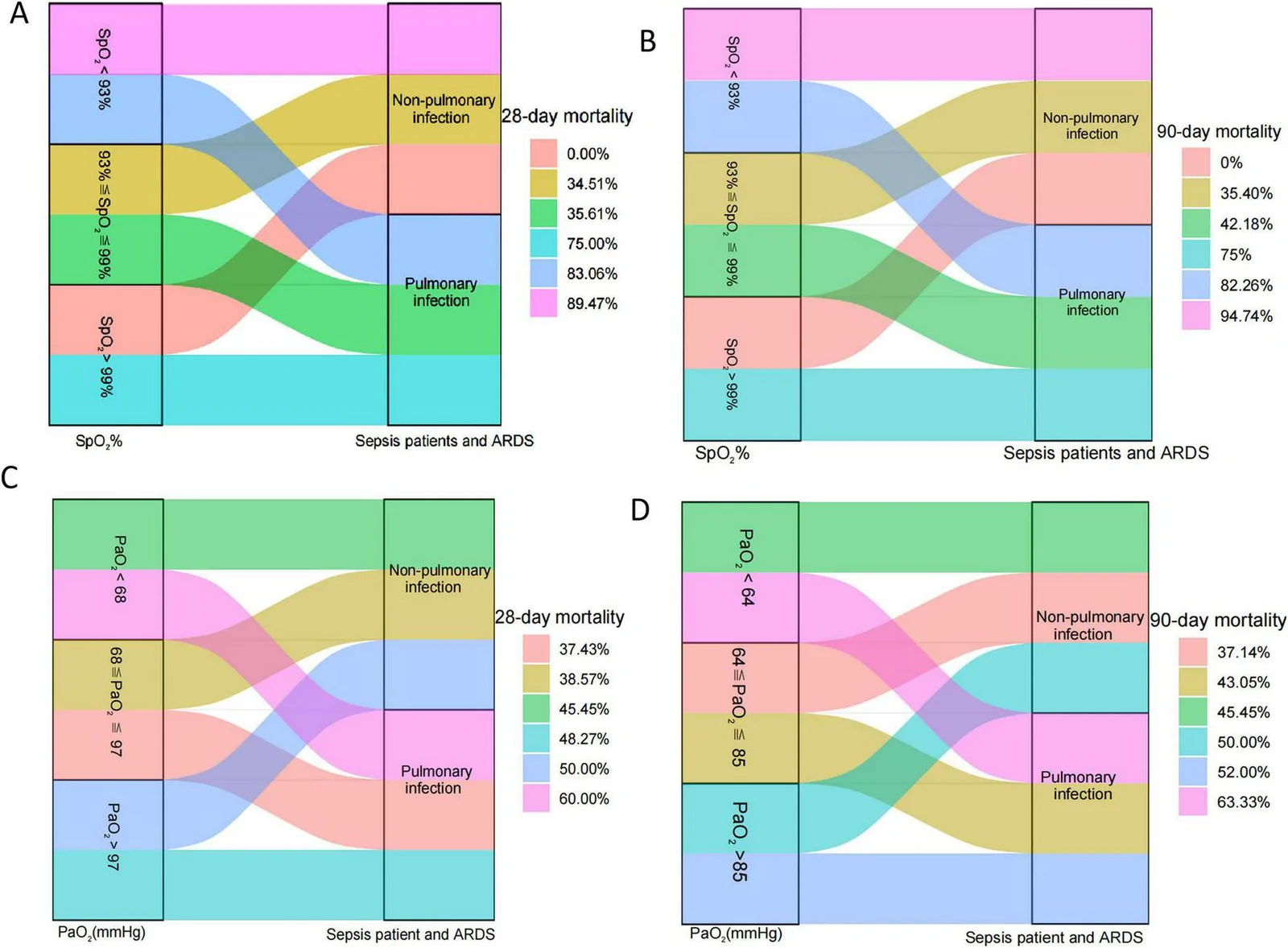
Stratified analysis of 28-day and 90-day mortality based on SpO_2_ **(A,B)** and PaO_2_ **(C,D)** in ARDS patients with and without pulmonary infection in Tianjin Chest Hospital Cohort.

**FIGURE 6:**
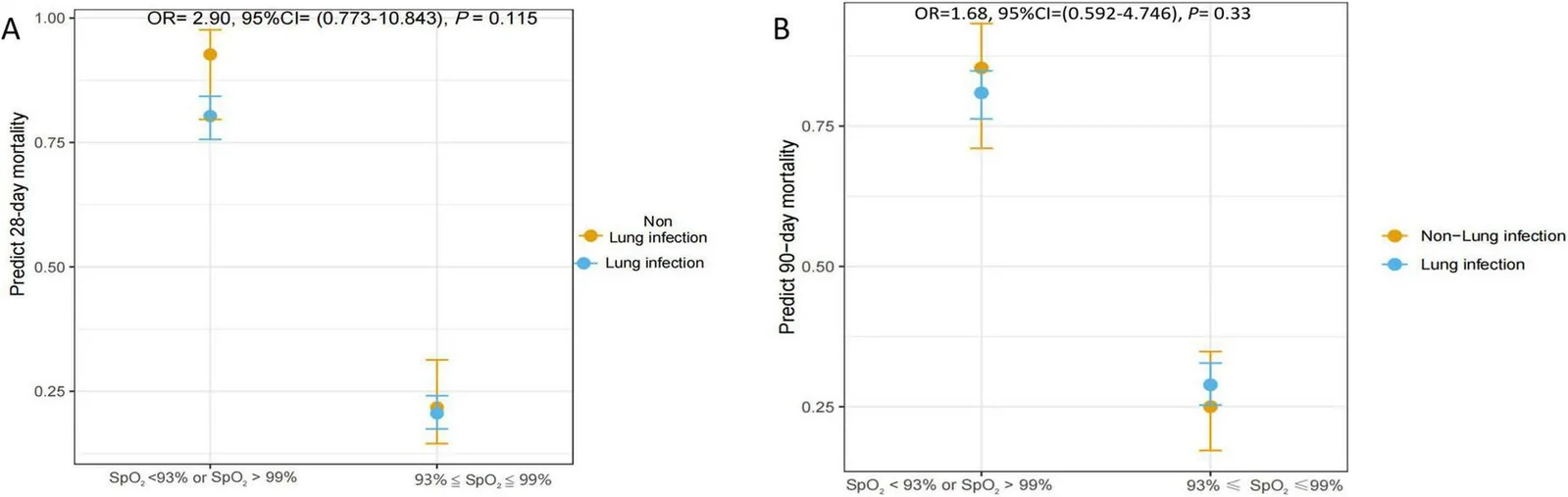
Subgroup analysis and interaction test for the association between SpO_2_ category and all cause mortality stratified by infection source (pulmonary vs. non-pulmonary sepsis) in patients with sepsis-associated ARDS. **(A)** Adjusted predicted 28-day mortality across SpO_2_ strata; **(B)** adjusted predicted 90-day mortality across SpO_2_ strata. The reference group was patients with SpO_2_ < 93% or SpO_2_ > 99%. Yellow circles and error bars denote patients with non-pulmonary infection; blue circles and error bars represent patients with pulmonary infection. Horizontal error bars indicate 95% confidence intervals for predicted mortality.

Figure 5 presents 28- and 90-day all-cause mortality stratified by SpO_2_ and PaO_2_ strata according to infection origin (Figures 5A–D). For non-pulmonary sepsis-related ARDS patients, those with SpO_2_ < 93% had higher 28- and 90-day mortality than patients with SpO_2_ of 93%–99% or SpO_2_ > 99%, while zero 28-day mortality was observed among non-pulmonary sepsis patients with SpO_2_ > 99%. In patients with pulmonary sepsis-associated ARDS, mortality was elevated in participants with SpO_2_ < 93% or SpO_2_ > 99% relative to those with SpO_2_ within 93%–99%. For 28-day outcomes, patients with PaO_2_ of 68–97 mmHg had lower mortality than individuals outside this range; elevated mortality beyond this PaO_2_ window was more prominent in patients with pulmonary infection. Similar patterns were identified for 90-day mortality when PaO_2_ fell outside 64–85 mmHg. We further performed interaction analyses to test effect modification by infection source. The interaction *P*-values were 0.115 (28-day mortality, OR = 2.90, 95% CI 0.773–10.843) and 0.33 (90-day mortality, OR = 1.68, 95% CI 0.592–4.746), indicating no significant interaction between SpO_2_ strata and infection source (Figure 6).

### A variety of statistical methods were used to verify the range of SpO_2_ value

The multivariate logistic analysis of the Tianjin Chest Hospital cohort showed that SpO_2_ levels between 93% and 99% were an independent correlate of lower 28-day all-cause mortality of patients with sepsis and ARDS [odds ratio (OR), 0.25; 95% confidence interval [CI], 0.18–0.36]. The accuracy of the study results was determined by the propensity match score. After matching the 10 variables listed in Supplementary eFigure 1A, propensity score matching (OR, 0.21; 95% CI, 0.11–0.37), propensity score IPW (OR, 0.11; 95% CI, 0.06–0.21), and doubly robust estimation with all covariates (OR, 0.59; 95% CI, 0.53–0.65) showed that SpO_2_ levels between 93% and 99% were an independent correlate of lower 28-day all-cause mortality of patients with sepsis and ARDS (Table 2 and Supplementary eTable 4).

The multivariate logistic analysis of the MIMIC IV Database (OR, 0.66; 95% CI, 0.45–0.98) and the eICU database (OR, 0.18; 95% CI, 0.12–0.25) showed that SpO_2_ levels between 93% and 99% were an independent correlate of lower 28-day all-cause mortality of patients with sepsis and ARDS (Table 2 and Supplementary eTables 6, 7). We comprehensively assessed propensity score balance via Supplementary eFigure 2 (MIMIC-IV) and Supplementary eFigure 3 (eICU-CRD): the IPTW weight histograms (Supplementary eFigures 2B, 3B) presented right-skewed weight distributions without extreme outliers; PSM weight plots (Supplementary eFigures 2C, 3C) displayed concentrated weights near the median, reflecting stable matching performance; kernel density curves of propensity scores (Supplementary eFigures 2D, 3D) demonstrated clear baseline risk separation between 28-day survivors and non-survivors.

Stabilized IPTW weights were calculated for all three cohorts, with no truncation applied to retain full weight distribution transparency. For the Tianjin Chest Hospital cohort, the mean IPTW weight was 2.021 (variance = 9.864), with weights ranging from 1.000 to 47.159; effective sample sizes (ESS) were 194.7 for the treated group and 135.6 for the control group, representing >80% of the original sample size. For the MIMIC-IV cohort, mean IPTW weight was 1.989 (variance = 9.733), with weights from 1.000 to 53.190; ESS was 86.0 (treated) and 634.4 (control), also >80% of the original sample. For the eICU-CRD cohort, mean IPTW weight was 1.987 (variance = 2.519), with weights from 1.003 to 12.991; ESS was 185.8 (treated) and 528.8 (control), with minimal sample dilution. Matching weight diagnostics for all cohorts showed acceptable distributions, with mean weights between 0.294–0.488 and variances <0.13, no negative weights, and maximum weights capped at 1.000. No extreme outlier weights were identified that would invalidate the stability of weighting or matching analyses (Supplementary eTable 9).

After matching the 14 variables from Supplementary eFigure 2A and 18 variables from Supplementary eFigure 3A, the propensity score IPW (OR, 0.67; 95% CI, 0.46–0.99) and the doubly robust method with all covariates (OR, 0.73; 95% CI, 0.58–0.90) showed that SpO_2_ levels between 93% and 99% were an independent correlate of lower 28-day all-cause mortality of patients with sepsis and ARDS in the MIMIC IV database (Table 2). Similarly, propensity score matching (OR, 0.68; 95% CI, 0.52–0.90), propensity score IPW (OR, 0.55; 95% CI, 0.39–0.77) and the doubly robust method with all covariates (OR, 0.67; 95% CI, 0.55–0.82) showed that SpO_2_ levels between 93% and 99% were an independent correlate of lower 28-day all-cause mortality of patients with sepsis and ARDS in the eICU database (Table 2). Notably, propensity score matching analysis for the MIMIC-IV cohort returned a non-significant association (OR = 0.88, 95% CI 0.64–1.22, *P* = 0.453), whereas the IPW and doubly robust models still identified that the 93%–99% SpO_2_ range was significantly correlated with lower 28-day mortality in this database (Table 2).

### The relationship between SpO_2_ levels of 93% to 99% and prognostic score

**FIGURE 7:**
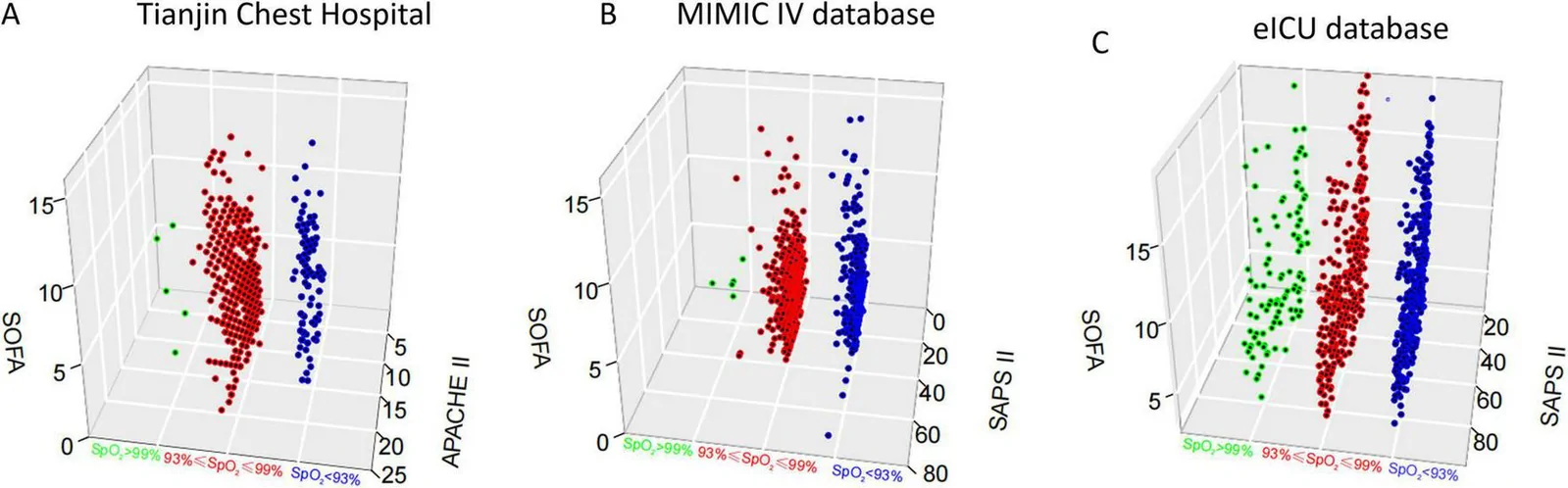
The relationship between SpO_2_ and prognostic scores in the three cohorts. **(A)** Shows the relationship between SpO_2_ and SOFA and APACHE II scores in the Tianjin Chest Hospital cohort. **(B,C)** Show the relationship between SpO_2_ and SOFA and SAPS II scores in the MIMIC IV and eICU databases. Green: SpO_2_ > 99%; red: 93% ≤ SpO_2_ ≤ 99%; blue: SpO_2_ < 93%.

Figures 7A–C show that the SOFA (*P* < 0.01 vs. *P* = 0.02), APACHE II (*P* < 0.01), and SAPS II (*P* = 0.01) scores for patients with sepsis and ARDS with SpO_2_ levels < 93% were significantly higher than those with SpO_2_ levels between 93% and 99% and SpO_2_ > 99% in the Tianjin Chest Hospital and MIMIC IV databases. Figure 4C shows that the SOFA and SAPS II levels for different SpO_2_ levels had no significant difference in the eICU database (Supplementary eTable 8).

### Stratified analyses by ARDS severity

**FIGURE 8:**
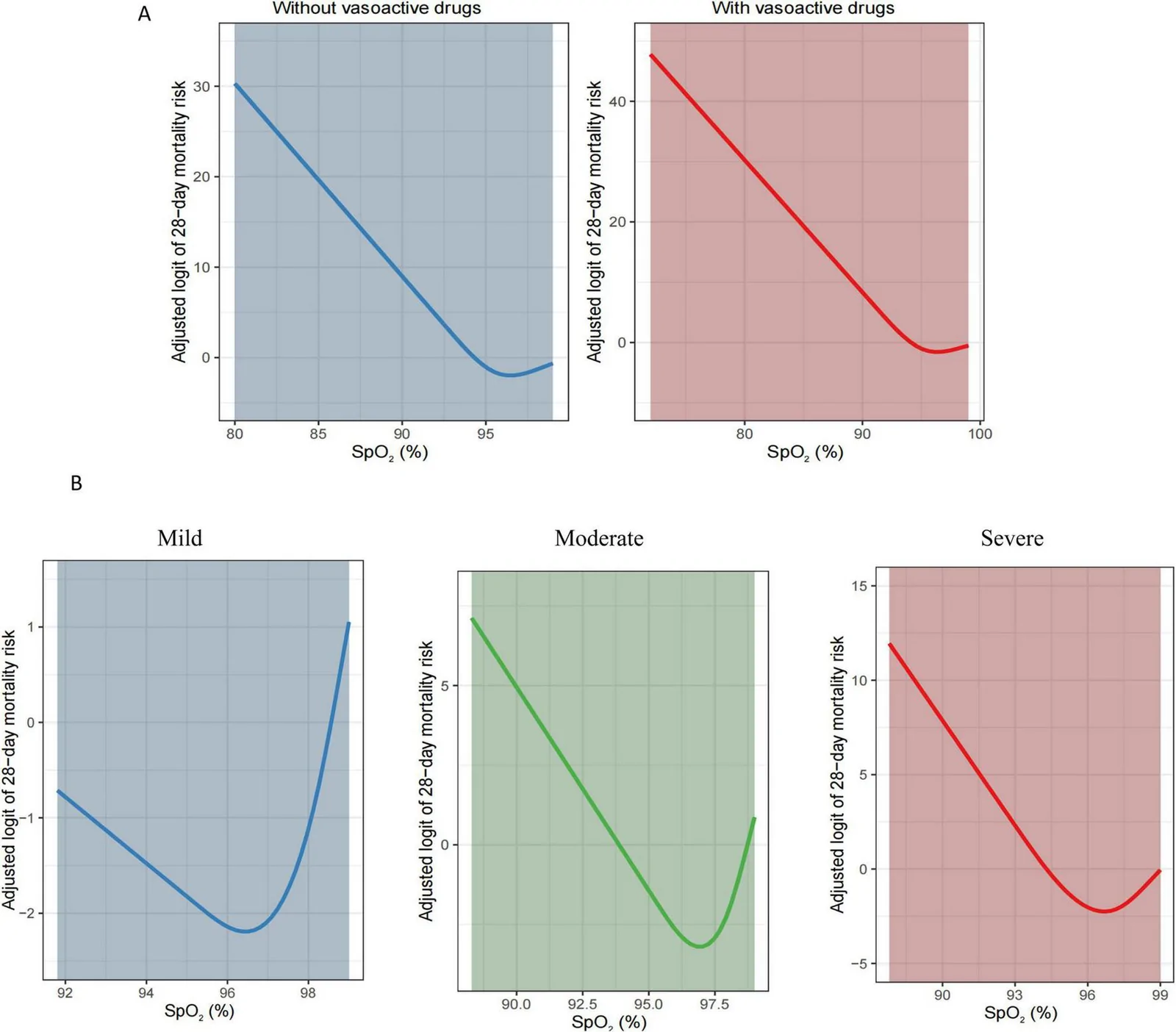
Fully adjusted restricted cubic spline (RCS) curves illustrating the nonlinear association between median SpO_2_ and adjusted logit of 28-day all-cause mortality risk, stratified by clinical subgroups. **(A)** Stratification by vasoactive agent use. Blue curve = patients without vasoactive support; red curve = patients receiving vasoactive drugs. A consistent U-shaped association is observed, with mortality minimized within the 93%–99% SpO_2_ window for both subgroups. **(B)** Stratification by Berlin ARDS severity grades. Blue = mild ARDS, green = moderate ARDS, red = severe ARDS. Across all severity strata, mortality risk reaches its nadir at SpO_2_ 93%–99%, with elevated risk below 93% and approaching 99%. All spline models were adjusted for SOFA score, APACHE II score, malignancy history, mechanical ventilation duration, and vasoactive/inotropic therapy. Solid colored lines denote predicted mortality logit; unshown shaded bands represent 95% confidence intervals.

Restricted cubic spline regression was applied to assess the non-linear relationship between SpO_2_ and adjusted logit of 28-day mortality risk in ARDS patients stratified by vasoactive drug use and ARDS severity, with particular focus on the clinically relevant SpO_2_ range of 93%–99%; in Figure 8A, for both patients with and without vasoactive drugs, mortality risk gradually decreased when SpO_2_ rose into the 93%–99% window, reached the minimal mortality risk within this interval near 95%, and started to slightly rebound toward the upper bound of 99%, while patients receiving vasoactive drugs consistently had a higher adjusted logit of 28-day mortality across the entire 93%–99% SpO_2_ range compared with those not administered vasoactive agents, and risk values remained markedly elevated at SpO_2_ below 93% in both subgroups; in Figure 8B, this pattern was reproducible across mild, moderate, and severe ARDS subgroups within the 93%–99% SpO_2_ range, where mild ARDS exhibited declining mortality risk from 93% to a nadir between 96% and 98% before risk elevation approaching 99%, moderate ARDS showed continuous risk reduction across 93% up to an optimal value near 97% with a mild rebound toward 99%, severe ARDS presented the highest baseline mortality logit at SpO_2_ starting at 93%, experienced prominent risk decline to its minimum around 96% within this range, and modest risk increase as SpO_2_ approached 99%, with baseline mortality risk at 93% following the gradient severe ARDS > moderate ARDS > mild ARDS, collectively demonstrating that SpO_2_ levels outside the 93%–99% range were associated with elevated 28-day mortality, and the 93%–99% saturation interval harbored a risk-minimizing plateau, with mortality risk lowest at intermediate SpO_2_ values within this window and rising at both the lower end below 93% and upper end approaching 99%.

### Distribution of TWA-SpO_2_ and SpO_2_-AUC across cohort

**FIGURE 9:**
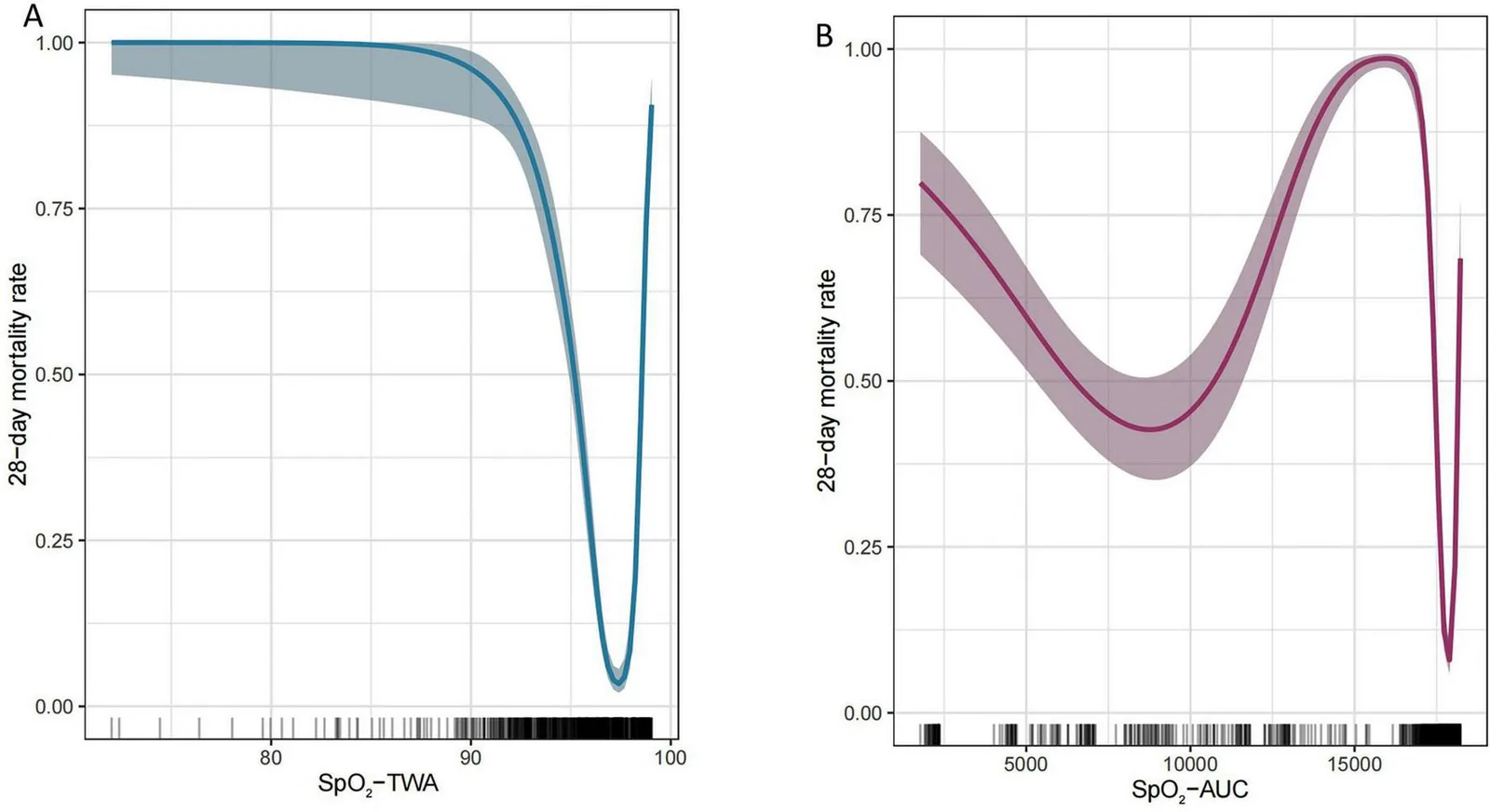
Restricted cubic spline regression curves showing adjusted non-linear associations between time-integrated oxygen exposure metrics and 28-day all-cause mortality in the Tianjin Chest Hospital sepsis-associated ARDS cohort, added in response to peer review suggestions. **(A)** Association between time-weighted average SpO_2_ (TWA-SpO_2_) and adjusted logit of 28-day mortality risk. A clear U-shaped relationship is observed, with mortality risk minimized when TWA-SpO_2_ falls within the 93%–99% interval. Solid blue line = predicted adjusted mortality logit; gray shaded band = 95% confidence interval; rug ticks along the *x*-axis represent individual patient TWA-SpO_2_ distribution. **(B)** Association between SpO_2_-time area under the curve (SpO_2_-AUC) and adjusted logit of 28-day mortality risk. A biphasic curve demonstrates the lowest mortality risk at SpO_2_-AUC values corresponding to sustained SpO_2_ of 93%–99% during mechanical ventilation. Solid magenta line = predicted adjusted mortality logit; light purple shaded band = 95% confidence interval; rug ticks along the *x*-axis denote individual patient SpO_2_-AUC distribution. All spline models were fully adjusted for SOFA score, APACHE II score, history of malignant tumor, duration of mechanical ventilation, and vasoactive/inotropic drug support.

We additionally modeled two physiologically relevant cumulative oxygen exposure endpoints (TWA-SpO_2_ and SpO_2_-AUC), with fully adjusted restricted cubic spline curves presented in Figure 9.

For TWA-SpO_2_ (Figure 9A), a pronounced U-shaped relationship with 28-day mortality was identified. Mortality risk gradually declined as TWA-SpO_2_ rose from low values, reaching its nadir within the 93%–99% TWA-SpO_2_ window; mortality risk steeply rebounded when TWA-SpO_2_ exceeded 99%, while substantially elevated mortality persisted at TWA-SpO_2_ below 93%. The minimal 28-day mortality risk plateau aligned exactly with our primary median SpO_2_ range of 93%–99%.

For SpO_2_-AUC (Figure 9B), the spline curve exhibited a biphasic relationship with 28-day all-cause mortality. Mortality risk initially decreased with rising SpO_2_-AUC; mortality risk progressively increased at intermediate SpO_2_-AUC values, before a sharp secondary risk reduction at the highest SpO_2_-AUC strata. Collectively, both time-weighted and area-under-curve integrated oxygen metrics validated our core finding: sustained oxygen saturation within 93%–99% over the full mechanical ventilation period conferred the lowest 28-day mortality risk for sepsis-associated ARDS patients.

## Discussion

Our data analysis showed a U-shaped relationship between SpO_2_ and the 28-day mortality rate of patients with sepsis and ARDS. The 28-day mortality rate was the lowest when the median SpO_2_ was between 93% and 99%.

This study found that the 28-day mortality rate for patients with sepsis and ARDS at Tianjin Chest Hospital was 42.6%. Patients with sepsis are prone to developing ARDS and have a high mortality rate, especially those on mechanical ventilation (4, 5). Our findings are consistent with previous studies that found a high incidence of patients with sepsis and ARDS.(4, 5, 18, 19) In addition, the definition of ARDS has recently been updated, (20, 21) highlighting that ARDS is not well understood, not effectively treated, and still associated with a high mortality rate. Therefore, effective prevention and control strategies for ARDS need to be explored.

Mechanically ventilated patients with ARDS require oxygen therapy, and pulmonary damage can be caused by high or low oxygen range (7). However, the range of oxygen for patients with ARDS has not yet been determined. A previous study found no difference in 28-day mortality between patients with ARDS on mechanical ventilation with SpO_2_ of 88%–92% and those with SpO_2_ ≥ 96% (8). The study included patients with ARDS from all causes. Among critically ill patients on mechanical ventilation, randomized trials have not identified oxygenation ranges that affect overall outcomes (22, 23). However, a recent study found that individualized oxygenation ranges could reduce mortality in critically ill adults, particularly those with sepsis (9). Based on these findings, we explored the range oxygenation values for patients with sepsis and ARDS.

We observed a robust U-shaped association between median SpO_2_ and 28-day mortality across our single-center cohort and two external ICU databases, with the lowest mortality occurring at 93%–99% SpO_2_ after multivariate logistic regression, PSM, IPW and doubly robust adjustment. However, this correlation cannot be taken as causal proof for this oxygen range, with disease severity acting as a critical confounding factor. Consistent with our subgroup analyses, patients with SpO_2_ < 93% exhibited markedly higher baseline SOFA, APACHE II and SAPS II scores, meaning hypoxemia may simply serve as an accompanying marker of critical organ dysfunction rather than an independent factor linked to elevated mortality risk. Oxygenation fluctuates dynamically during ventilation and is heavily confounded by progressive disease severity. Although we adjusted for baseline single-time-point severity scores via multivariable regression, propensity score matching, IPW and doubly robust models, our static analytical framework fails to capture time-varying organ dysfunction and dynamic fluctuations in illness severity over the entire ventilation course. Hypoxemia below 93% or hyperoxia above 99% may only signal severe underlying illness, while SpO_2_ values within 93%–99% may only correlate with stable physiological status instead of representing a modifiable correlate linked to reduced mortality. These methodological limitations restrict causal interpretation of the observed SpO_2_-mortality association, as the apparent survival difference across SpO_2_ strata may be partially or fully driven by baseline and evolving disease severity. These methodological limitations restrict causal interpretation of the observed oxygenation-mortality relationship. Mortality increased markedly in patients with SpO_2_ < 93% or >99%, as well as those receiving FiO_2_ above 60%. In contrast, multiple divergent PaO_2_ optimal intervals were only observed in single-center internal models, and none of these PaO_2_ thresholds achieved significant survival benefits in external validation cohorts. All PaO_2_-related results should be interpreted as exploratory rather than actionable clinical ranges. Collectively, our data demonstrate that SpO_2_ values within 93%–99% correlate with the lowest mortality risk in mechanically ventilated sepsis-associated ARDS patients, consistent with prior real-world critical care oxygenation research, consistent with prior critical care oxygenation studies (9).

Our observational results show that a SpO_2_ range of 93%–99% correlates with improved survival among patients with sepsis-associated ARDS receiving mechanical ventilation. This conclusion differs from a prior RCT enrolling unselected ARDS patients, which may be attributable to disparate predefined oxygenation ranges and absence of sepsis-specific subgroup analyses in that trial. Two large observational ICU cohorts previously reported favorable outcomes with SpO_2_ ranges of 95%–99% and 94%–98%, and our 93%–99% SpO_2_ window aligns closely with these real-world findings (24, 25). Patients with SpO_2_ < 93% exhibited substantially higher SOFA, APACHE II, and SAPS II scores, signifying greater disease severity; this risk gradient was most pronounced among patients with non-pulmonary sepsis. Notably, while multiple PaO_2_ cutoffs were generated from internal GAM and RCS models, these intervals failed external validation and cannot guide clinical oxygen titration. Only the 93%–99% SpO_2_ range demonstrated consistent survival protection across all datasets and analytical frameworks, and this narrow SpO_2_ band merits evaluation in future prospective oxygenation trials for sepsis-ARDS. The overstated benefits from PSM/IPW result from matched sample truncation and volatile propensity weights. Combining propensity weighting and full outcome regression, doubly robust estimation minimizes confounding and model misspecification bias, so we regard its moderate inverse correlational association as primary evidence, which was replicated in MIMIC-IV and eICU-CRD cohorts. Despite satisfactory covariate balance (all SMD < 0.1), unmeasured time-varying severity confounders partly explain cross-method discrepancies.

We supplemented static median SpO_2_ analyses with two time-integrated oxygen load indicators: TWA-SpO_2_ and SpO_2_-AUC. Unlike a single snapshot median value, TWA-SpO_2_ and SpO_2_-AUC capture cumulative, time-dependent hypoxemia/hyperoxia burden across the entire mechanical ventilation course, better reflecting real-world dynamic oxygen titration at the bedside. Both integrated metrics recapitulated our primary conclusion: sustained oxygen saturation confined to the 93%–99% range over ventilatory support independently correlates with the lowest 28-day mortality risk. The U-shaped TWA-SpO_2_ curve confirmed that cumulative prolonged hypoxemia (TWA-SpO_2_ < 93%) and chronic hyperoxic exposure (TWA-SpO_2_ > 99%) each drive elevated mortality risk. The biphasic SpO_2_-AUC distribution further illustrated that intermediate cumulative hyperoxia load carries excess mortality risk, while stable long-term SpO_2_ within 93%–99% minimizes cumulative oxygen-related lung and end-organ injury. These time-weighted analyses eliminate bias from static single-time-point saturation summaries and reinforce the generalizability of our proposed 93%–99% SpO_2_ target for mechanically ventilated sepsis-ARDS patients.

Notably, despite decades-long evolution in mechanical ventilation, sepsis treatment and oxygenation guidance, the SpO_2_ window of 93%–99% remained consistently correlated with better survival in both pre-pandemic U.S. retrospective databases and a modern post-COVID Chinese cohort. This temporal consistency suggests the harmful effects of tissue hypoxia (SpO_2_ < 93%) and pulmonary hyperoxic injury (SpO_2_ > 99%) are consistent across evolving ICU care frameworks, reinforcing the generalizability of oxygen ranges for mechanically ventilated sepsis-ARDS patients regardless of era-specific ventilator or sepsis management routines.

From a real-world ICU implementation perspective, our 93%–99% SpO_2_ range carries practical operational implications for bedside oxygen titration. Continuous pulse oximetry alarms can be configured with lower thresholds near 93% to prompt timely FiO_2_ escalation to avoid sustained hypoxemia, while upper alarm limits set at 99% can alert clinicians to excessive oxygen delivery and potential hyperoxic lung injury. During routine ventilator titration, clinicians need coordinated adjustment of FiO_2_ and PEEP to maintain saturation within this window: patients with borderline SpO_2_ should first receive PEEP optimization before raising FiO_2_ to minimize high oxygen exposure. Institutional standardized oxygen titration protocols built around this saturation range may reduce inconsistent bedside oxygen management. Still, individual physiological variability persists–patients with severe chronic lung disease or refractory hypoxemia may require temporary SpO_2_ above 99% as a rescue measure, necessitating personalized protocol exceptions to balance safety and respiratory support needs. These operational details strengthen the translational utility of our oxygen range for routine critical care practice.

The PaO_2_-mortality association failed external validation, largely due to cross-cohort disparities in arterial blood gas sampling frequency, unsynchronized ABG timing, inconsistent FiO_2_ levels and divergent ventilation protocols. Unlike regularly recorded SpO_2_, irregular, clinically driven PaO_2_ measurements lack uniform steady-state normalization across databases, introducing substantial heterogeneity that weakens its reproducible prognostic value.

This study has several limitations. First, we recognize a critical limitation regarding time-varying ICU confounders. Only admission-only SOFA, APACHE II and SAPS II scores were available for adjustment; we lacked daily serial SOFA, fluctuating vasopressor doses and real-time severity metrics during ventilation. Despite multiple static statistical adjustments, residual confounding from evolving organ dysfunction and hemodynamic instability remains, preventing full separation of the SpO_2_–mortality association from dynamic illness severity. Future studies incorporating longitudinal severity data and time-varying analytical models are needed to address this issue. Second, key lung-protective ventilation variables (tidal volume, plateau pressure, driving pressure, prone positioning) were unadjusted in all regression, propensity score, IPW, and double-robust models due to severe missing and inconsistently recorded data across cohorts. These parameters are critical confounders linking SpO_2_ and ARDS mortality: elevated tidal volume/driving pressure worsen ventilator-induced lung injury, while prone positioning ameliorates hypoxemia and survival, each independently altering oxygenation and clinical outcomes. Omitting these metrics introduces residual confounding that may bias the magnitude of the SpO_2_–28-day mortality association. Though PEEP was uniformly documented, it was also excluded from primary adjustment. Unmeasured confounders such as ventilator mode and sedation depth further weaken causal inference for our 93%–99% SpO_2_ range. Importantly, the survival benefit of this SpO_2_ window was reproducible across three geographically and temporally separate ICU cohorts despite this limitation. Prospective studies with standardized, complete ventilation data are required to definitively validate this oxygenation target and eliminate residual bias. Third, we also acknowledge a limitation related to our SpO_2_ aggregation strategy. We summarized serial oxygen measurements into a single median value across the full ventilation window, which masks intra-individual temporal fluctuations and cannot distinguish differential associations between SpO_2_ and mortality during early versus late ventilation phases. Without time-stratified SpO_2_ exposure metrics, we could not evaluate whether the SpO_2_–mortality link varies across distinct stages of respiratory support, leaving phase-specific effects uncharacterized. Future analyses using time-partitioned oxygen data or time-weighted exposure indices are required to resolve this issue. Our static median SpO_2_ exposure summary may induce immortal time bias and fails to reflect real-time oxygen fluctuations corresponding to routine clinical decision-making throughout ventilation. Fourth, another key limitation relates to our longitudinal oxygenation processing: we capped monitoring at day 7 and aggregated serial SpO_2_ into patient medians, obscuring within-patient oxygen dynamics and potentially causing informative censoring. Early and late ventilation phases differ markedly in lung injury and inflammation, which may modify the SpO_2_-mortality association. Cross-cohort inconsistencies in long-term ventilation records precluded phase-stratified analyses here. Another important limitation involves confounding by disease severity: patients with SpO_2_ outside 93%–99% had markedly higher baseline SOFA, APACHE II and SAPS II scores, creating strong confounding between abnormal saturation and worse survival. Although we adjusted for baseline severity via multiple statistical strategies, we lacked dynamic, time-varying severity metrics to fully isolate the SpO_2_ signal from progressive organ dysfunction. We thus cannot rule out that the apparent survival advantage of the 93%–99% SpO_2_ group mainly stems from milder underlying illness, rather than the saturation range itself. Prospective trials with serial severity assessments are needed to resolve this confounding. Fifth, we only adjusted for single-time-point baseline severity scores (SOFA, APACHE II, SAPS II) rather than dynamic longitudinal severity indicators, such as daily SOFA trajectories and serial vasopressor dose escalation over mechanical ventilation. These time-varying markers of progressive organ dysfunction may induce residual confounding that cannot be fully eliminated by our static propensity score and regression models, which limits our ability to fully distinguish whether low SpO_2_ independently predicts mortality or merely reflects worsening underlying disease severity. Sixth, temporal heterogeneity across cohorts constitutes another limitation. MIMIC-IV (2008–2019) and eICU-CRD (2014–2015) reflect pre-COVID US ICU practice, while our single-center cohort (2023–2026) represents contemporary Chinese critical care. Evolving ventilation, sepsis and oxygenation protocols may weaken cross-cohort comparability. Still, the 93%–99% SpO_2_ range showed consistent prognostic benefit across all datasets, implying this association is robust to era-specific clinical variations. Finally, race/ethnicity subgroup analyses were not conducted due to severe sample imbalance across racial categories in MIMIC-IV and eICU-CRD, limiting our ability to evaluate the potential confounding impact of skin pigmentation-related SpO_2_ bias on the 93%–99% saturation threshold’s external validity. Further large, racially balanced datasets are required to address this issue in subsequent research.

## Conclusion

This hypothesis-generating retrospective multi-database analysis identifies a consistent association between SpO_2_ 93%–99% and lower 28-day all-cause mortality in mechanically ventilated sepsis-associated ARDS patients across three independent ICU cohorts. Given the inherent limitations of observational research including residual confounding from unmeasured ventilation parameters and time-varying illness severity, these correlational findings cannot establish causality and should not be adopted as definitive clinical oxygenation targets. Our results only generate a testable hypothesis for future prospective randomized controlled trials to verify the clinical utility of this SpO_2_ window.

## Data Availability

The raw data supporting the conclusions of this article will be made available by the authors, without undue reservation.

## Supplementary material

Supplementary eFigure 1 Propensity score diagnostic plots evaluating covariate balance, inverse probability weighting (IPTW) distribution, matching weight distribution, and propensity score density distribution stratified by 28-day survival status in the Tianjin Chest Hospital cohort. (A) Absolute standardized mean difference (SMD) plot for all included baseline covariates before (red circles, unadjusted) and after propensity score adjustment (cyan triangles, adjusted). The vertical dashed line denotes the conventional balance threshold at SMD = 0.1; SMD < 0.1 indicates adequate intergroup covariate balance. (B) Histogram showing the distribution of individual IPTW weights. Red dashed line marks the weight median. Y-axis represents participant count, X-axis denotes IPTW weight magnitude. (C) Histogram of weight distribution after propensity score matching (PSM). Red dashed line corresponds to weight median; Y-axis = number of subjects, X-axis = matching weight. (D) Kernel density plot illustrating propensity score distribution between survivors (pink shaded area) and non-survivors (cyan shaded area) at 28 days. X-axis = estimated propensity score value, Y-axis = probability density.

Supplementary eFigure 2 Propensity score diagnostic plots assessing covariate balance, IPTW distribution, PSM weight distribution, and propensity score density stratified by 28-day mortality in the MIMIC-IV cohort. (A) Absolute standardized mean difference plot for baseline covariates before (red circles, unadjusted) and after propensity score adjustment (blue triangles, adjusted). Vertical dashed line at SMD = 0.1 as the cutoff for acceptable covariate balance. (B) Histogram of individual IPTW weight distribution, with red dashed line indicating median weight; X-axis = IPTW weight, Y-axis = subject count. (C) Histogram of post-matching weight distribution for propensity score matching, red dashed line for median weight; X-axis = matching weight, Y-axis = participant count. (D) Kernel density curve comparing propensity score distribution between 28-day survivors (pink) and non-survivors (cyan). X-axis = propensity score, Y-axis = probability density.

Supplementary eFigure 3 Propensity score diagnostic graphs verifying covariate balance, IPTW weight distribution, PSM weight distribution, and group-specific propensity score density stratified by 28-day survival outcome in the eICU-CRD cohort. (A) Absolute standardized mean difference of all baseline confounders pre-adjustment (red circles) and post-adjustment (cyan triangles). Vertical dashed line at SMD = 0.1 defines adequate covariate balance. (B) Histogram of IPTW weight distribution, red dashed line represents median weight; X-axis = IPTW weight value, Y-axis = number of patients. (C) Histogram of propensity score matching weight distribution, red dashed line for median weight; X-axis = matching weight, Y-axis = patient count. (D) Kernel density plot of propensity scores separated by 28-day survival (pink) and non-survival (cyan). X-axis = propensity score, Y-axis = probability density.

Supplementary eFigure 4 Flow chart. ARDS:Acute respiratory distress syndrome; MIMIC IV: Medical Information Mart for Intensive Care IV;eICU-CRD:eICU Collaborative Research Database.

Supplementary eFigure 5 The relationship between blood oxygen index and 90-day mortality of patients with sepsis and ARDS during mechanical ventilation therapy in Tianjin Chest Hospital. Solid red lines = predicted mortality logit; dashed red lines = 95% confidence intervals; horizontal dotted line = baseline reference risk; vertical dashed lines = critical oxygen thresholds; x-axis rug ticks represent individual patient data distribution.

Supplementary eFigure 6 Kaplan–Meier (GAM) 28-day survival analyses stratified by PaO_2_ cut-off values in external validation cohorts, accompanied by number-at-risk tables. (A) MIMIC-IV cohort; (B) eICU-CRD cohort. Red = non-optimal PaO_2_ group; cyan = optimal PaO_2_ group. X-axis = follow-up days; Y-axis = cumulative survival probability.

Supplementary eFigure 7 Kaplan–Meier (RCS) 28-day survival analyses stratified by different PaO_2_ cut-off values in eICU-CRD and MIMIC-IV cohorts, with number-at-risk tables. (A) eICU-CRD, PaO_2_ < 81 or > 110 mmHg vs. 81–110 mmHg; (B) MIMIC-IV, PaO_2_ < 81 or > 110 mmHg vs. 81–110 mmHg; (C) eICU-CRD, PaO_2_ < 80 or > 90 mmHg vs. 80–90 mmHg; (D) MIMIC-IV, PaO_2_ < 80 or > 90 mmHg vs. 80–90 mmHg. Red: non-optimal PaO_2_ group; cyan: optimal PaO_2_ group. X-axis = follow-up days; Y-axis = cumulative survival probability.

Supplementary eTable 2 Baseline data on 28-day mortality in sepsis patients and ARDS in Tianjin Chest Hospital.

Supplementary eTable 9 Propensity Score Diagnostics for Three Independent Cohorts.
