# Supplementary material for: Association between oxygen saturation ranges and mortality among mechanically ventilated sepsis-associated ARDS patients: a multicenter retrospective cohort analysis
Source: Front Med (Lausanne). 2026 Aug 21;13:1862391. doi: 10.3389/fmed.2026.1862391 (PMC13541905; doi:10.3389/fmed.2026.1862391)
Supplement: Supplementary file 1 [file Table_1.docx]

**eTable 1** Missing rates of key covariates across three study cohorts

| **Variables** | **Tianjin Chest Hospital** | **MIMICIV** | **eICU** |
| --- | --- | --- | --- |
| Respiratory rate | 1.1% | 1.5% | 1.2% |
| SOFA / APACHE II / SAPS II scores | 4.6%–7.2% | 6.3%–9.5% | 5.1%–8.7% |
| Coagulation indexes | 2.5%–5.3% | 4.2%–6.9% | 3.0%–6.1% |
| Hepatic function markers | 3.1%–6.8% | 4.7%–8.1% | 3.4%–7.5% |
| Renal function markers | 2.8%–6.2% | 4.0%–7.6% | 3.2%–6.9% |
