# Supplementary material for: Association between oxygen saturation ranges and mortality among mechanically ventilated sepsis-associated ARDS patients: a multicenter retrospective cohort analysis
Source: Front Med (Lausanne). 2026 Aug 21;13:1862391. doi: 10.3389/fmed.2026.1862391 (PMC13541905; doi:10.3389/fmed.2026.1862391)
Supplement: Supplementary file 2 [file Table_2.docx]

| **eTable 2** Baseline data on 28-day mortality in sepsis patients and ARDS in Tianjin Chest Hospital | | | |
| --- | --- | --- | --- |
| **Characteristic** | Survivor(*n*=587) | Non-Survivor(*n*=435) | *P* |
| **Co-morbid conditions, n (%)** | | | |
| Hypertension | 322 ( 54.9) | 239 ( 54.9) | 1.000 |
| Diabetes | 196 ( 33.4) | 124 ( 28.5) | 0.110 |
| Chronic obstructive pulmonary disease | 114 ( 19.4) | 79 ( 18.2) | 0.669 |
| Cardiovascular | 186 ( 31.7) | 137 ( 31.5) | 1.000 |
| Hepatic | 75 ( 12.8) | 67 ( 15.4) | 0.268 |
| Chronic kidney diseae | 132 ( 22.5) | 108 ( 24.8) | 0.425 |
| Metastatic cancer | 91 ( 15.5) | 101 ( 23.2) | 0.002 |
| **Physiology** | | | |
| Temperature, °C | 37.30[36.50,38.30] | 36.90 [36.50, 38.20] | 0.047 |
| Heart rate, beats per minute | 105.00[87.00,120.50] | 109.00[88.00,126.50] | 0.098 |
| Systolicblood pressure, mmHg | 119.00 [100.50, 143.00] | 114.00 [87.00, 140.00] | 0.003 |
| Diastolicblood pressure, mmHg | 62.00 [51.00, 75.00] | 60.00 [45.00, 75.00] | 0.004 |
| **Coagulation function** | | | |
| Platelet (×10ˆ9 /L) | 134.00 [70.00, 209.00] | 103.00 [43.50, 169.00] | <0.001 |
| Hemoglobin(g/dL) | 86.00 [68.00, 108.00] | 81.00 [66.00, 109.00] | 0.142 |
| PT(sec) | 13.10 [11.90, 14.70] | 13.70 [12.10, 16.15] | <0.001 |
| INR | 1.20 [1.09, 1.34] | 1.26 [1.11, 1.46] | 0.001 |
| APTT(sec) | 30.80 [27.65, 34.40] | 31.60 [28.10, 37.15] | 0.006 |
| FIB(g/L) | 3.72 [2.83, 4.80] | 3.66 [2.64, 4.62] | 0.086 |
| **Liver function** | | | |
| ALT(U/L) | 32.00 [18.00, 60.50] | 34.00 [20.00, 84.00] | 0.126 |
| AST(U/L) | 47.00 [30.00, 95.50] | 59.00 [32.50, 131.50] | 0.001 |
| Albumin(g/L) | 31.00 [29.00, 32.00] | 31.00 [26.00, 33.00] | 0.040 |
| Total Bilirubin(umol/L) | 14.30 [9.30, 24.15] | 16.10 [10.25, 26.90] | 0.015 |
| **Kidney function** | | | |
| Creatinine(umol/L) | 138.00 [71.00, 267.00] | 172.00 [89.50, 319.50] | 0.003 |
| Bun (mmol/L) | 14.10 [8.00, 21.00] | 16.00 [9.00, 23.00] | 0.028 |
| **Cardiac function** | | | |
| CK(U/L) | 222.00[82.00,603.50] | 276.00[96.50,867.50] | 0.008 |
| CK-MB(U/L) | 22.00 [12.00, 51.00] | 29.00 [14.50, 90.00] | <0.001 |
| **Indicators of infection** |  |  |  |
| WBCx10ˆ9/L | 13.32[9.52, 19.38] | 14.08[9.31, 20.28] | 0.603 |
| CRP(mg/L) | 10.85 [3.99, 20.00] | 16.80 [5.50, 20.00] | 0.001 |
| PCT(ng/mL) | 2.37 [0.76, 8.49] | 3.19 [1.11, 10.89] | 0.015 |
| **The internal environment of the organism** | | | |
| Sodium(mmol/L) | 141.00 [136.30, 145.50] | 141.00 [136.00, 147.80] | 0.365 |
| Potassium(mmol/L) | 4.56 [3.89, 5.12] | 4.61 [3.87, 5.42] | 0.157 |
| Glucose(mmol/L) | 12.00 [8.60, 16.75] | 11.90 [8.10, 16.80] | 0.505 |

ALT: Alanine aminotransferase; APPT: Activated partial thrombin time; AST: Aspartate aminotransferase; BUN: blood urea nitrogen; CK: Creatine Kinase; CK-MB: Creatine kinase myocardial band; CRP: C-reactive protein; FIB: Fibrinogen; INR: International Normalized Ratio; PCT: Procalcitonin; PT: Prothrombin time; WBC: White blood cell; *p* < 0.05, statistically significant.
