# Supplementary material for: Association between oxygen saturation ranges and mortality among mechanically ventilated sepsis-associated ARDS patients: a multicenter retrospective cohort analysis
Source: Front Med (Lausanne). 2026 Aug 21;13:1862391. doi: 10.3389/fmed.2026.1862391 (PMC13541905; doi:10.3389/fmed.2026.1862391)
Supplement: Supplementary file 3 [file Table_3.docx]

| **eTable3** Baseline data on 90-day mortality in sepsis patients and ARDS in Tianjin Chest Hospital | | | |
| --- | --- | --- | --- |
| **Characteristic** | Survivor (*n*=538) | Non-Survivor(*n*=484) | *P* |
| Age, years | 67.00 [53.00, 75.00] | 68.00 [58.00, 75.00] | 0.051 |
| Male sex, n (%) | 221 (41.1) | 172 (35.5) | 0.079 |
| **Co-morbid conditions, n (%)** | | | |
| Hypertension | 289 (53.7) | 272 (56.2) | 0.464 |
| Diabetes | 181 (33.6) | 139 (28.7) | 0.104 |
| Chronic obstructive pulmonary disease | 112 (20.8) | 81 (16.7) | 0.113 |
| Cardiovascular | 169 (31.4) | 154 (31.8) | 0.943 |
| Hepatic | 75 (13.9) | 67 (13.8) | 1.000 |
| Chronic kidney diseae | 131 (24.3) | 109 (22.5) | 0.539 |
| Immunosuppression by disease | 72 (13.4) | 57 (11.8) | 0.498 |
| Metastatic cancer | 85 (15.8) | 107 (22.1) | 0.013 |
| **Site of infection, n (%)** | | | |
| Lung | 463 (86.1) | 426 (88.0) | 0.404 |
| Abdominal | 121 (22.5) | 94 (19.4) | 0.261 |
| Urinary | 51 (9.5) | 26 (5.4) | 0.018 |
| Skin softtissue | 24 (4.5) | 15 (3.1) | 0.332 |
| Catheter | 27 (5.0) | 28 (5.8) | 0.687 |
| **Pathogenic microorganisms, n (%)** | | | |
| Acinetobacter baumannii | 78 (14.5) | 123 (25.4) | <0.001 |
| Klebsiella pneumoniae | 94 (17.5) | 152 (31.4) | <0.001 |
| Pseudomonas aeruginosa | 70 (13.0) | 50 (10.3) | 0.218 |
| Escherichiacoli | 48 (8.9) | 37 (7.6) | 0.532 |
| Staphylococcus aureus | 55 (10.2) | 57 (11.8) | 0.488 |
| **Physiology** | | | |
| Temperature, °C | 37.40 [36.50, 38.30] | 36.90 [36.50, 38.23] | 0.040 |
| Heart rate, beats per minute | 105.00 [87.25, 121.00] | 108.00 [88.00, 125.00] | 0.149 |
| Systolicblood pressure, mmHg | 119.00 [100.00, 142.00] | 117.00 [89.00, 140.00] | 0.055 |
| Diastolicblood pressure, mmHg | 62.00 [51.00, 75.00] | 60.00 [46.00, 75.00] | 0.015 |
| **Indicators of infection** | | | |
| WBCx10ˆ9/L | 13.43 [9.60, 19.76] | 13.75 [8.95, 19.55] | 0.575 |
| CRP(mg/L) | 10.35 [3.64, 20.00] | 17.90 [5.60, 20.00] | <0.001 |
| PCT(ng/L) | 2.36 [0.73, 8.35] | 3.26 [1.14, 11.08] | 0.007 |
| **Coagulation function** |  |  |  |
| Platelet(×10ˆ9 /L) | 132.00[68.25, 207.00] | 108.50 [45.00, 176.25] | <0.001 |
| Hemoglobin(g/dL) | 86.00[68.00, 108.00] | 81.00 [66.00, 108.00] | 0.133 |
| PT(sec) | 13.05 [11.80, 14.70] | 13.70 [12.20, 16.02] | <0.001 |
| INR | 1.20 [1.09, 1.34] | 1.25 [1.12, 1.45] | 0.001 |
| APTT(sec) | 30.90 [27.70, 34.40] | 31.25 [28.00, 36.42] | 0.119 |
| FIB | 3.68 [2.84, 4.74] | 3.76 [2.65, 4.67] | 0.226 |
| **Liver function** |  |  |  |
| ALT(U/L) | 30.50 [18.00, 58.00] | 35.50 [21.00, 83.25] | 0.010 |
| AST(U/L) | 45.00 [29.00, 90.00] | 59.00 [34.00, 133.00] | <0.001 |
| Albumin(g/L) | 31.00 [29.00, 32.00] | 31.00 [26.00, 33.00] | 0.012 |
| Total Bilirubin(umol/L) | 14.25 [9.22, 23.25] | 16.05 [10.20, 27.90] | 0.009 |
| **Kidney function** |  |  |  |
| Creatinine (umol/L) | 138.50 [72.25, 267.00] | 165.00 [86.75, 315.25] | 0.030 |
| Bun (mmol/L) | 14.75 [8.43, 21.00] | 15.35 [9.00, 22.60] | 0.165 |
| **Cardiac function** |  |  |  |
| CK(U/L) | 222.00 [80.25, 600.50] | 262.50 [97.00, 853.75] | 0.014 |
| CK-MB(U/L) | 22.00 [12.00, 50.75] | 28.00 [14.00, 87.00] | 0.001 |
| **The internal environment of the organism** | | | |
| Sodium (mmol/L) | 141.10 [136.70, 145.40] | 140.90 [135.90, 147.85] | 0.905 |
| Potassium (mmol/L) | 4.60 [3.92, 5.18] | 4.54 [3.85, 5.31] | 0.791 |
| Glucose (mmol/L) | 12.20 [8.72, 16.88] | 11.65 [8.00, 16.60] | 0.087 |
| Lac_0 h | 1.80 [1.30, 3.00] | 2.40 [1.50, 4.60] | <0.001 |
| Lac_6 h | 1.80 [1.30, 2.98] | 2.40 [1.50, 4.00] | <0.001 |
| Lac_12 h | 1.70 [1.30, 2.60] | 2.30 [1.50, 3.50] | <0.001 |
| Lac_18 h | 1.60 [1.20, 2.40] | 2.10 [1.50, 3.12] | <0.001 |
| Lac_24 h | 1.60 [1.22, 2.28] | 2.10 [1.40, 3.10] | <0.001 |
| **Respiration** |  |  |  |
| Respiratory rate, breaths per minute | 23.00 [18.00, 30.00] | 24.00 [18.00, 30.00] | 0.537 |
| SpO_2_, % | 97.00 [94.00, 98.00] | 94.00 [92.00, 95.00] | <0.001 |
| PaO_2_, mmHg | 93.95 [88.10, 107.07] | 75.10 [67.57, 85.23] | <0.001 |
| PaCO_2_, mmHg | 37.00 [32.00, 42.55] | 37.00 [31.60, 44.00] | 0.899 |
| FiO_2_ | 0.50 [0.41, 0.60] | 0.60 [0.45, 0.70] | <0.001 |
| PaO_2_/FiO_2_ ratio, mmHg | 211.50 [154.83, 248.66] | 183.73 [127.46, 236.41] | <0.001 |
| SPO_2_/FiO_2_ , ratio | 186.00 [157.62, 235.00] | 158.33 [128.57, 206.67] | <0.001 |
| PEEP, cmH_2_O | 7.20 [6.60, 7.60] | 8.00 [7.50, 8.20] | <0.001 |
| **Therapy, *n* (%)** | | | |
| Prone ventilation therapy | 22 (4.1) | 17 (3.5) | 0.751 |
| Use of glucocorticoids | 116 (21.6) | 92 (19.0) | 0.350 |
| Use of muscle relaxant | 19 (3.5) | 12 (2.5) | 0.426 |
| **Outcomes** | | | |
| ICU length of stay (days) | 13.82 [8.00, 24.08] | 11.52 [4.83, 20.44] | <0.001 |
| Inotropic/vasopressor support, *n* (%) | 285 (53.0) | 356 (73.6) | <0.001 |
| Duration of mechanical ventilation, (days) | 7.00 [3.15, 14.40] | 8.00 [3.27, 15.49] | 0.507 |
| Renal replacement therapy, n (%) | 153 (28.4) | 216 (44.6) | <0.001 |
| APACHE II score | 14.00 [13.00, 17.00] | 24.00 [21.00, 26.00] | <0.001 |
| SOFA score | 7.00 [5.00, 8.00] | 10.00 [8.00, 13.00] | <0.001 |

ALT: Alanine aminotransferase; APACHE II score: Acute physiology and chronic health evaluation score; APPT: Activated partial thrombin time; AST: Aspartate aminotransferase; BUN: blood urea nitrogen; CK: Creatine Kinase; CK-MB: Creatine kinase myocardial band; CRP: C-reactive protein; FIB: Fibrinogen; FiO_2_: Fraction of inspiration O_2_; INR: International Normalized Ratio; Lac: Lactates; PaCO_2_: partial pressure of carbon dioxide; PaO_2_: partial pressure of oxygen; PCT: Procalcitonin; PEEP: positive end-expiratory pressure; PT: Prothrombin time; APACHE II:AcutePhysiology and Chronic Health Evaluation; SOFA: Sequential organ failure assessment; SpO_2_: arterial oxygen saturation; WBC: White blood cell; *P* < 0.05, statistically significant.
