# Supplementary material for: Association between oxygen saturation ranges and mortality among mechanically ventilated sepsis-associated ARDS patients: a multicenter retrospective cohort analysis
Source: Front Med (Lausanne). 2026 Aug 21;13:1862391. doi: 10.3389/fmed.2026.1862391 (PMC13541905; doi:10.3389/fmed.2026.1862391)
Supplement: Supplementary file 4 [file Table_4.docx]

**eTable 4** Multivariate logistic regression analysis of 28-day prognosis in patients with sepsis and ARDS in Tianjin Chest Hospital

| **Characteristics** | OR | 95% CI | *P*-value |
| --- | --- | --- | --- |
| Age | 1.01 | 1.00-1.01 | 0.11 |
| History_Cancer | 1.37 | 1.09-1.73 | 0.010 |
| Urinary infection | 0.61 | 0.39-0.95 | 0.030 |
| *Acinetobacter baumannii infection* | 1.04 | 0.82-1.31 | 0.760 |
| *Klebsiella pneumoniae infection* | 0.84 | 0.66-1.05 | 0.130 |
| *Pseudomonas aeruginosa infection* | 0.73 | 0.52-1.03 | 0.070 |
| CRP | 1.00 | 1.00-1.00 | 0.770 |
| PCT | 1.00 | 0.99-1.00 | 0.280 |
| FiO_2_ ≥ 63% | 1.74 | 0.9-3.35 | 0.100 |
| 68 ≦ PaO_2_ ≦ 97 | 0.72 | 0.57-0.89 | <0.001 |
| SpO_2_/FiO_2_ | 1.00 | 1.00-1.00 | 0.170 |
| PaO_2_/FiO_2_≥190 | 0.77 | 0.39-1.51 | 0.450 |
| SOFA | 0.93 | 0.89-0.96 | <0.001 |
| APACHE II | 1.21 | 1.17-1.24 | <0.001 |
| Vasopressor or inotropic support | 2.01 | 1.58-2.54 | <0.001 |
| CRRT treatment | 1.02 | 0.84-1.24 | 0.850 |

APACHE II score: Acute physiology and chronic health evaluation score; CRP: C-reactive protein; CRRT: Continuous Renal Replacement Therapy; FiO_2_: Fraction of inspiration O_2_; PaO_2_: partial pressure of oxygen; PCT: Procalcitonin; SOFA: Sequential organ failure assessment; SpO_2_: arterial oxygen saturation; *p* < 0.05, statistically significant.
