# Supplementary material for: Association between oxygen saturation ranges and mortality among mechanically ventilated sepsis-associated ARDS patients: a multicenter retrospective cohort analysis
Source: Front Med (Lausanne). 2026 Aug 21;13:1862391. doi: 10.3389/fmed.2026.1862391 (PMC13541905; doi:10.3389/fmed.2026.1862391)
Supplement: Supplementary file 5 [file Table_5.docx]

**eTable 5** The Specificity and Sensitivity of 28-day mortality in patients with sepsis and ARDS in Tianjin Chest Hospital

| **Characteristics** | **AUC** | **Specificity** | **Sensitivity** | ***P*-value** |
| --- | --- | --- | --- | --- |
| History_Cancer | 0.539 | 0.845 | 0.232 | 0.035 |
| PaO_2_< 68 or > 97 | 0.555 | 0.545 | 0.566 | 0.002 |
| SpO_2_<93 or >99 | 0.624 | 0.961 | 0.287 | <0.001 |
| APACHE II | 0.846 | 0.857 | 0.653 | <0.001 |
| Vasopressor or inotropic support | 0.630 | 0.777 | 0.484 | <0.001 |

APACHE II score: Acute physiology and chronic health evaluation score; PaO_2_: partial pressure of oxygen; SpO_2_: arterial oxygen saturation; *p* < 0.05, statistically significant.
