# Supplementary material for: Association between oxygen saturation ranges and mortality among mechanically ventilated sepsis-associated ARDS patients: a multicenter retrospective cohort analysis
Source: Front Med (Lausanne). 2026 Aug 21;13:1862391. doi: 10.3389/fmed.2026.1862391 (PMC13541905; doi:10.3389/fmed.2026.1862391)
Supplement: Supplementary file 6 [file Table_6.docx]

**eTable 6** Multivariate Logistic regression analysis of 28-day prognosis in sepsis patients and ARDS in MIMIC IV database

| **Characteristics** | **OR** | **95% CI** | *P-value* |
| --- | --- | --- | --- |
| Age | 1.026 | 1.010-1.043 | 0.002 |
| Sex | 1.525 | 1.034-2.249 | 0.033 |
| SOFA | 1.255 | 1.155-1.363 | <0.001 |
| SPAS II | 1.055 | 0.987-1.023 | 0.587 |
| Chronic kidney disease | 0.443 | 0.203-0.970 | 0.042 |
| Hepatic | 1.239 | 0.583-2.632 | 0.578 |
| Immunosuppression by disease | 4.115 | 2.344-7.225 | <0.001 |
| Heart rate | 1.009 | 0.999-1.018 | 0.070 |
| Platelet | 1.004 | 1.002-1.006 | <0.001 |
| PT | 1.023 | 0.953-1.099 | 0.523 |
| INR | 1.059 | 0.495-2.268 | 0.882 |
| APTT | 0.995 | 0.987-1.003 | 0.251 |
| Creatinine | 1.525 | 1.059-2.198 | 0.023 |
| Bun | 1.035 | 1.012-1.058 | 0.003 |

APPT: Activated partial thrombin time; BUN: blood urea nitrogen; INR: International Normalized Ratio; PT: Prothrombin time; SAPSII: simplified acute physiology score; SOFA: Sequential organ failure assessment; *p* < 0.05, statistically significant.
