# Supplementary material for: Association between oxygen saturation ranges and mortality among mechanically ventilated sepsis-associated ARDS patients: a multicenter retrospective cohort analysis
Source: Front Med (Lausanne). 2026 Aug 21;13:1862391. doi: 10.3389/fmed.2026.1862391 (PMC13541905; doi:10.3389/fmed.2026.1862391)
Supplement: Supplementary file 7 [file Table_7.docx]

**eTable 7** Multivariate Logistic regression analysis of 28-day prognosis in sepsis patients and ARDS in eICU database

| **Characteristics** | **OR** | **95% CI** | *P-value* |
| --- | --- | --- | --- |
| Age | 1.009 | 0.998-1.020 | 0.093 |
| Cardiovascular | 1.817 | 1.111-2.969 | 0.017 |
| Chronic obstructive pulmonary disease | 2.085 | 1.343-3.237 | 0.001 |
| Chronic kidney disease | 1.204 | 0.709-2.045 | 0.492 |
| Abdominal infection | 1.664 | 0.848-3.264 | 0.139 |
| Temperature | 0.993 | 0.723-1.363 | 0.965 |
| Heart rate | 1.003 | 0.991-1.016 | 0.607 |
| Diastolic blood pressure | 1.005 | 0.996-1.014 | 0.296 |
| WBC | 1.014 | 0.996-1.033 | 0.122 |
| Platelet | 1.000 | 0.998-1.001 | 0.558 |
| PT | 0.943 | 0.885-1.005 | 0.071 |
| INR | 1.691 | 0.928-3.081 | 0.086 |
| Creatinine | 0.804 | 0.697-0.928 | 0.003 |
| Bun | 1.008 | 0.999-1.018 | 0.090 |
| Glucose | 0.999 | 0.997-1.001 | 0.232 |
| Lac | 1.076 | 1.013-1.144 | 0.018 |
| SOFA | 1.239 | 1.173-1.309 | <0.001 |
| SAPS II | 0.993 | 0.987-1.000 | 0.061 |
| ICU length of stay | 0.955 | 0.930-0.981 | 0.001 |
| Vasoactive | 1.209 | 0.839-1.743 | 0.309 |

BUN: blood urea nitrogen; INR: International Normalized Ratio; Lac: Lactates; PT: Prothrombin time; SAPSII: simplified acute physiology score; SOFA: Sequential organ failure assessment; SpO_2_: arterial oxygen saturation; WBC: White blood cell; *p* < 0.05, statistically significant.
