# Supplementary material for: Association between oxygen saturation ranges and mortality among mechanically ventilated sepsis-associated ARDS patients: a multicenter retrospective cohort analysis
Source: Front Med (Lausanne). 2026 Aug 21;13:1862391. doi: 10.3389/fmed.2026.1862391 (PMC13541905; doi:10.3389/fmed.2026.1862391)
Supplement: Supplementary file 8 [file Table_8.docx]

| **eTable 8** Relationship between SpO_2_ and prognostic score in sepsis patients and ARDS | | | | |
| --- | --- | --- | --- | --- |
|  | SpO_2_ > 99% | 93% ≦ SpO_2_≦ 99% | SpO_2_ < 93% | *P* |
| **Tianjin Chest Hospital** | | | | |
| SOFA | 8.00[4.00, 11.00] | 8.00 [6.00, 11.00] | 10.00 [8.00, 12.00] | <0.001 |
| APACHE II | 18.00[17.00,20.00] | 18.00 [13.00, 24.00] | 24.00[18.25,25.00] | <0.001 |
| **MIMIC IV database** | | | | |
| SOFA | 4.00[4.00, 4.00] | 4.00 [3.00, 6.00] | 5.00 [3.00, 7.00] | 0.002 |
| SAPS II | 27.00[26.00,28.00] | 33.00 [26.00, 40.00] | 36.00[28.00,45.00] | 0.001 |
| **eICU database** | | | | |
| SOFA | 8.00 [6.00, 11.00] | 8.00 [6.00, 11.00] | 8.00 [6.00, 10.00] | 0.092 |
| SAPS II | 57.00[30.00,78.00] | 52.00 [30.00, 75.00] | 51.00[30.00,70.00] | 0.253 |

APACHE II score: Acute physiology and chronic health evaluation score; SAPSII: simplified acute physiology score; SOFA: Sequential organ failure assessment; *p* < 0.05, statistically significant.
